# Mitochondrial quality control: a new mechanism for antiviral therapy

**DOI:** 10.3389/fimmu.2026.1798516

**Published:** 2026-03-11

**Authors:** Xujie Duan, Wenjing Lan, Rui Liu, Pei Zhang, Sixu Chen, Yufei Zhang, Liang Zhang, Huiping Li, Shuying Liu

**Affiliations:** 1 College of Veterinary Medicine, Inner Mongolia Agricultural University, Hohhot, China; 2 Inner Mongolia Key Laboratory of Veterinary Fundamentals and Disease Control of Herbivorous livestock, Inner Mongolia Agricultural University, Hohhot, China; 3 Inner Mongolia Key Laboratory of Basic Veterinary Medicine, Inner Mongolia Agricultural University, Hohhot, China

**Keywords:** mitochondrial biogenesis, mitochondrial dynamics, mitochondrial quality control, mitophagy, viral infection

## Abstract

Mitochondria are highly dynamic organelles involved in energy production, metabolic regulation, calcium homeostasis, apoptosis, and innate immunity. The mitochondrial network is susceptible to damage from physiological and environmental factors, including viral infections. Mitochondrial quality control (MQC) is the primary pathway that maintains normal physiological functions and mitochondrial homeostasis. Mitochondrial dynamics and mitophagy are complex processes within the MQC mechanism that can be exploited by viruses to modulate mitochondrial morphology, metabolism, and innate immune responses, achieving immune evasion, promoting self-replication, and accelerating infection. Viruses or their proteins target mitochondrial dynamics or mitophagy and regulate these processes via direct or indirect mechanisms. In addition, numerous molecular modulators of MQC have been reported. These findings provide new opportunities to understand the MQC process and have the potential for use as antiviral therapeutic agents. This article reviews the relationships between MQC, viral infection events, and viral pathogenesis, introduces the known molecular pharmacological regulators of MQC, and emphasizes their importance in antiviral drug development.

## 1 Background

Mitochondria are multifunctional organelles within eukaryotic cells that play essential roles in cellular energy metabolism, signal transduction, apoptosis, and the regulation of host innate immune responses. The mitochondrial network is vulnerable to physiological and environmental insults, making the maintenance of functional homeostasis crucial for effective resistance against pathogenic infections. Mitochondria sense pathogen invasion and internal damage through alterations in mitochondrial membrane structure and protein expression, which can result in mitochondrial dysfunction and, consequently, affect cellular metabolism, oxidative stress, proliferation, and apoptosis. The integrity of the mitochondrial network is preserved through complex mitochondrial quality control (MQC) mechanisms (1).

MQC constitutes an endogenous protective mechanism that maintains mitochondrial homeostasis and physiological functions through three primary processes: mitochondrial biogenesis (formation, growth, and replication), dynamics (systematic regulation of fusion and fission), and mitophagy (recruitment of autophagy-related molecules to form mitophagosomes and selective lysosomal degradation) (2). The equilibrium between mitochondrial fission and fusion is central to organelle quality control, influencing and coordinating cellular metabolism and complex signaling cascades, thus participating in the regulation of cell pluripotency, division, differentiation, aging, and death. Fusion events allow for the compensation of compromised mitochondrial function by combining damaged mitochondria, whereas fission separates damaged mitochondrial regions, facilitating their targeted degradation via mitophagy. Accordingly, the coordinated actions of fission, fusion, and mitophagy prevent the propagation of defective mitochondria within the healthy mitochondrial pool (3). Furthermore, MQC moderates excessive mitochondrial-mediated immune activation and abnormal immune signaling, thus circumventing excessive inflammatory responses. Signals generated by the innate mitochondrial immune response provide feedback regulation for MQC, facilitating a concerted cellular response to stress. Many viruses interfere with MQC mechanisms and exploit these processes to enhance viral replication and evade host immune defenses (1).

Viral replication induces significant physiological changes in host cells, many of which disrupt the mitochondrial network and precipitate mitochondrial dysfunction. Hence, a growing body of research has examined how viral infections affect host mitochondrial function and immunity, and the role of MQC in the pathogenesis of viral infections. Many viruses manipulate the balance of mitochondrial fusion, fission, and mitophagy to regulate immune status and apoptosis, thereby promoting their replication, assembly, and dissemination and the development of infection-related pathologies.

This review seeks to comprehensively delineate the role of MQC in viral infections, with particular emphasis on how viral manipulation of mitochondrial biogenesis, dynamics, and mitophagy subverts host defenses and advances persistent infection and disease progression. This article summarizes the latest literature on mitochondrial functional alterations caused by viral infections, aiming to support foundational research in this evolving field.

## 2 Mitochondria and viral infection

### 2.1 Mitochondrial structure and function

Mitochondria are essential metabolic hubs within cells, distinguished by their highly organized structure and compartmentalized functions. Structurally, mitochondria comprise the outer mitochondrial membrane (OMM), intermembrane space (IMS), inner mitochondrial membrane (IMM), and matrix (4). Among them, the OMM is porous, facilitating the exchange of ions and transport of small molecules via porins. Membrane proteins, such as voltage-dependent anion channel protein 1 (VDAC1), mediate the transfer of ions and uncharged small molecules, whereas specific translocases manage the transport of macromolecules (5). The OMM also regulates several signaling pathways.

The IMS participates in protein transport and modification, redox state regulation, and cellular metabolism (6). The IMM is a tightly sealed diffusion barrier with selective permeability, permitting the passage of specific ions and molecules through specialized transport proteins and functioning as the primary site for oxidative phosphorylation (OXPHOS) (7). The cristae, formed by the inward folding of IMM, are unique structures that house most of the electron transport chain components and adenosine triphosphate (ATP) synthase, serving as the primary site of energy conversion in mammalian cells. The morphology and density of cristae adapt dynamically to cellular energy demands. The mitochondrial matrix supports numerous enzymatic reactions, DNA replication, transcription, and protein biosynthesis. It also generates the transmembrane electrochemical gradient required for ATP synthesis (6).

During oxidative respiration, mitochondria generate energy, which is stored as a proton gradient across the IMM, thereby establishing the mitochondrial membrane potential (MMP) (8). MMP stability is essential for optimal OXPHOS and ATP production, directly affecting the functions and health status of cells. Decreased MMP can trigger physiological responses, such as apoptosis and mitophagy (9).

**Figure 1:**
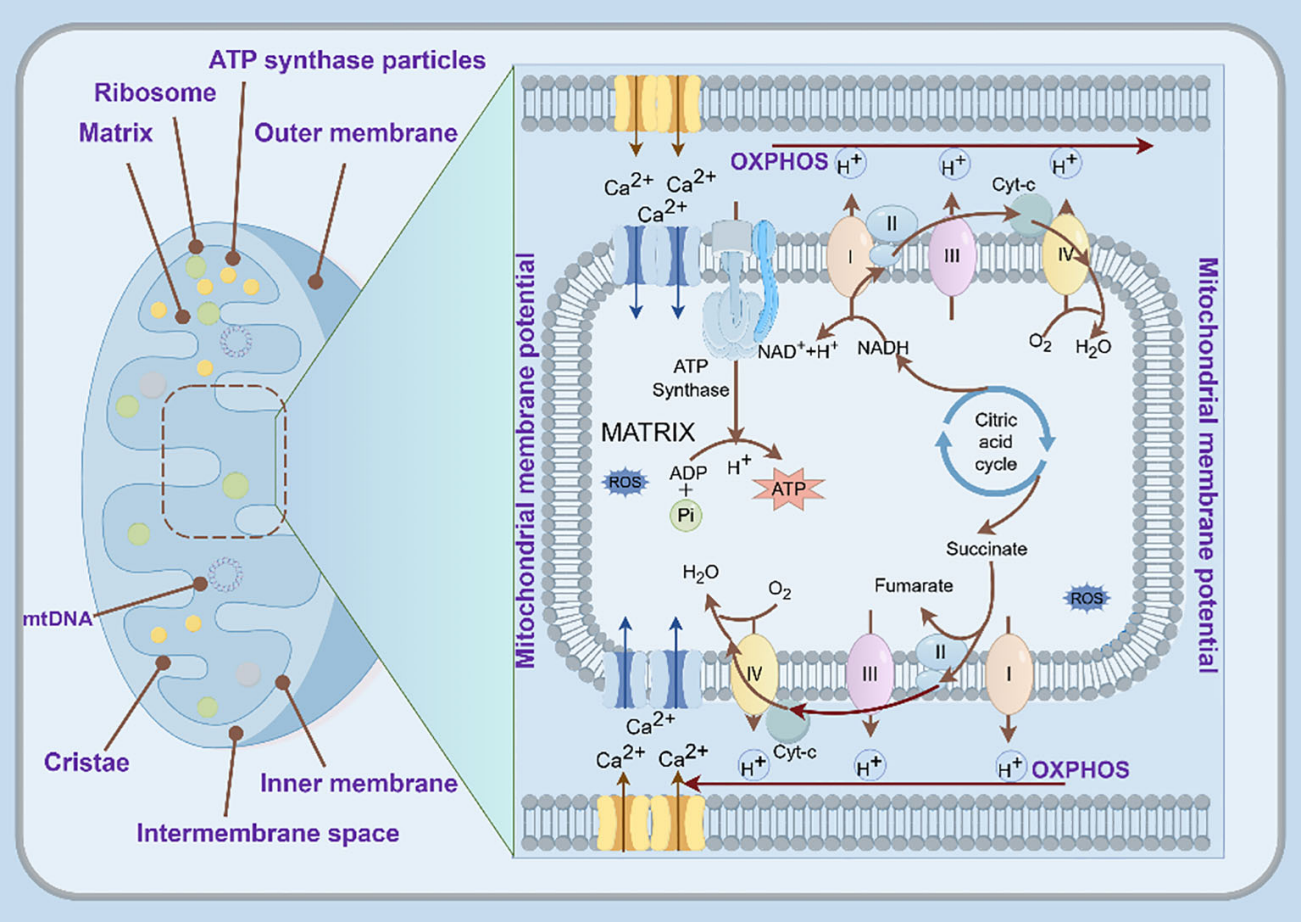
Basic structure and function of mitochondria. The diagram shows IMM house respiratory supercomplexes (I–IV) that shuttle electrons (brown arrows) while pumping protons outward, building an electrochemical gradient. The resulting proton-motive force drives ATP synthase to make ATP. ATP, adenosine triphosphate; IMM, inner mitochondrial membrane; OXPHOS, oxidative phosphorylation (By Figdraw).

Moreover, disruptions in mitochondrial aerobic metabolism or electron transport chain activity, and reactive oxygen species (ROS) generated during enzymatic reactions, significantly influence signal transduction and cellular homeostasis (**Figure 1**). The sensitivity of the electron transport chain to ROS positions mitochondria as particularly susceptible to oxidative damage (10). Mitochondrial DNA (mtDNA) is replicated and transcribed within the organelle, contributing to the assembly of the IMM respiratory chain, energy provision for cells, and mammalian embryonic development.

### 2.2 Effects of viral infection on mitochondria

Viruses lack intrinsic mechanisms for energy metabolism and must invade living host cells to function. They depend on host cell metabolic byproducts, including nucleotides, amino acids, and fatty acids, to supply energy, biosynthetic precursors for viral protein synthesis and mature virion assembly, as well as to manipulate host metabolism by reorganizing organelle structure. Viruses hijack cellular organelles, including the mitochondria, endoplasmic reticulum (ER), lipid droplets, and cytoskeleton, to promote their replication (11). During infection, viruses induce physiological changes in mitochondria, including disruptions in calcium homeostasis, ER stress, oxidative stress, and hypoxia, altering the cellular environment and leading to mitochondrial dysfunction and changes in MMP. These alterations result in increased production of mitochondrial ROS (mtROS) and the release of mitochondrial mtDNA and calcium ions (Ca^2+^). Through the release of pro-apoptosis factors such as cytochrome C (Cyt-C), mitochondria mediate endogenous apoptosis pathways (5). To promote the survival of infected cells, viruses have evolved strategies to inhibit apoptosis, including the manipulation of mitophagy and mitochondrial dynamics to remove damaged mitochondria (12).

**Figure 2:**
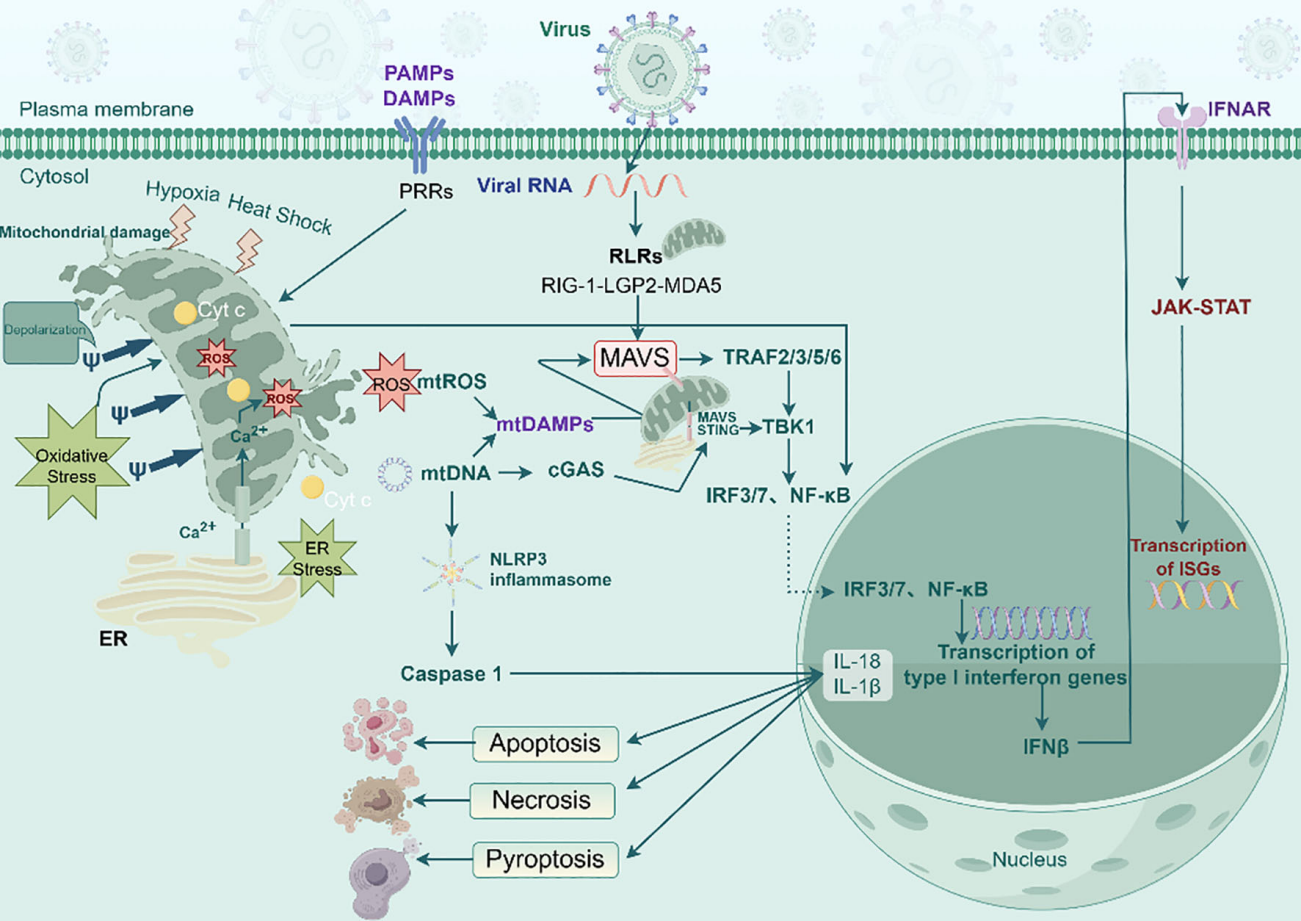
Effects of viral infection on mitochondria. mtDNA released due to mitochondrial damage caused by viral infection and viral RNA are captured by cGAS or RIG-I, respectively; both signals converge on the OMM adaptor MAVS. MAVS prion-like aggregation recruits TNF receptor-associated factors (TRAFs) and TBK1, triggering IRF3 phosphorylation and IFN-β transcription. Simultaneously, viral engagement collapses MMP, amplifying ROS and NLRP3 inflammasome activation, linking pathogen sensing, organelle stress, and antiviral defense. cGAS, cGAMP synthase; DAMPs, damage-associated molecular patterns; IFN, interferon; IRF, interferon regulatory factor; MAVS, mitochondrial antiviral signaling protein; MMP, mitochondrial membrane potential; mt, mitochondrial; NLRP3, NOD-, LRR-, and pyrin domain-containing protein 3; OMM, outer mitochondrial membrane; PAMP, pathogen-associated molecular pattern molecule; RLR, RIG1-like receptor; ROS, reactive oxygen species; STING, stimulator of interferon genes; TBK1, TANK-binding kinase 1 or TNF-α-activated protein kinase; TRAF, TNF receptor-associated factor (By Figdraw).

Hosts mount antiviral responses by remodeling organelles, with mitochondria serving as targets for viral proteins seeking to suppress host defense and as a key hub for metabolic regulation and innate immune signaling (13). During viral infection, pattern recognition receptors (PRRs), such as Toll-like receptors (TLRs), NOD-like receptors (NLRs), and C-type lectin receptors, first detect pathogen-associated molecular patterns and mitochondrial damage-associated molecular patterns (mtDAMPs). Upon activation, cellular metabolism shifts from OXPHOS to glycolysis, enabling rapid energy generation to combat viral infection (14). Activation of multiple intracellular signaling cascades results in the downstream nuclear translocation of transcription factors, nuclear factor kappa-light-chain-enhancer of activated B cells (NF-κB), interferon regulatory factor (IRF)3, and IRF7, which drives the expression of pro-inflammatory cytokines, including interferons (IFNs) (15). Secreted IFNs bind to their respective cell surface receptors, triggering the Janus kinase (JAK)–signal transducer and activator of transcription (STAT) pathway and stimulating the expression of numerous IFN-stimulated genes that defend against pathogenic microorganisms. Consequently, mitochondria are central targets of PRR-driven antiviral responses (16). The OMM also mediates antiviral immunity via retinoic acid inducible gene 1 (RIG-1)-like receptors (RLRs), including RIG-1, melanoma differentiation-associated gene 5 (MDA5), and laboratory of genetics and physiology 2 (LGP2). RIG-I and MDA5 are typical PRRs, whereas LGP2 is a regulator of RIG-I and MDA5 signal transduction (17). The mitochondrial antiviral signaling protein (MAVS), an RLR adaptor protein located on the OMM, senses viral RNA through RLRs. After activation by upstream RIG-I/MDA5, MAVS forms prion-like aggregates, initiating downstream signaling that activates IRF3 and NF-κB, leading to IFN-β expression and inhibition of viral replication. mtDNA and mtROS released following mitochondrial damage facilitate MAVS complex assembly, activate the NOD-, LRR-, and pyrin domain-containing protein 3 (NLRP3) inflammasome, and play a pivotal role in mitochondrial-mediated innate immunity (18). Furthermore, mtDNA can trigger the cyclic guanosine monophosphate-adenosine monophosphate (cGAMP) synthase (cGAS)–stimulator of interferon genes (STING) pathway, in which cGAS senses and recognizes cytoplasmic mtDNA, catalyzes the production of cGAS, and activates STING, leading to increased expression of IFNs and other immune molecules (19) (**Figure 2**).

## 3 MQC mechanisms

**Figure 3:**
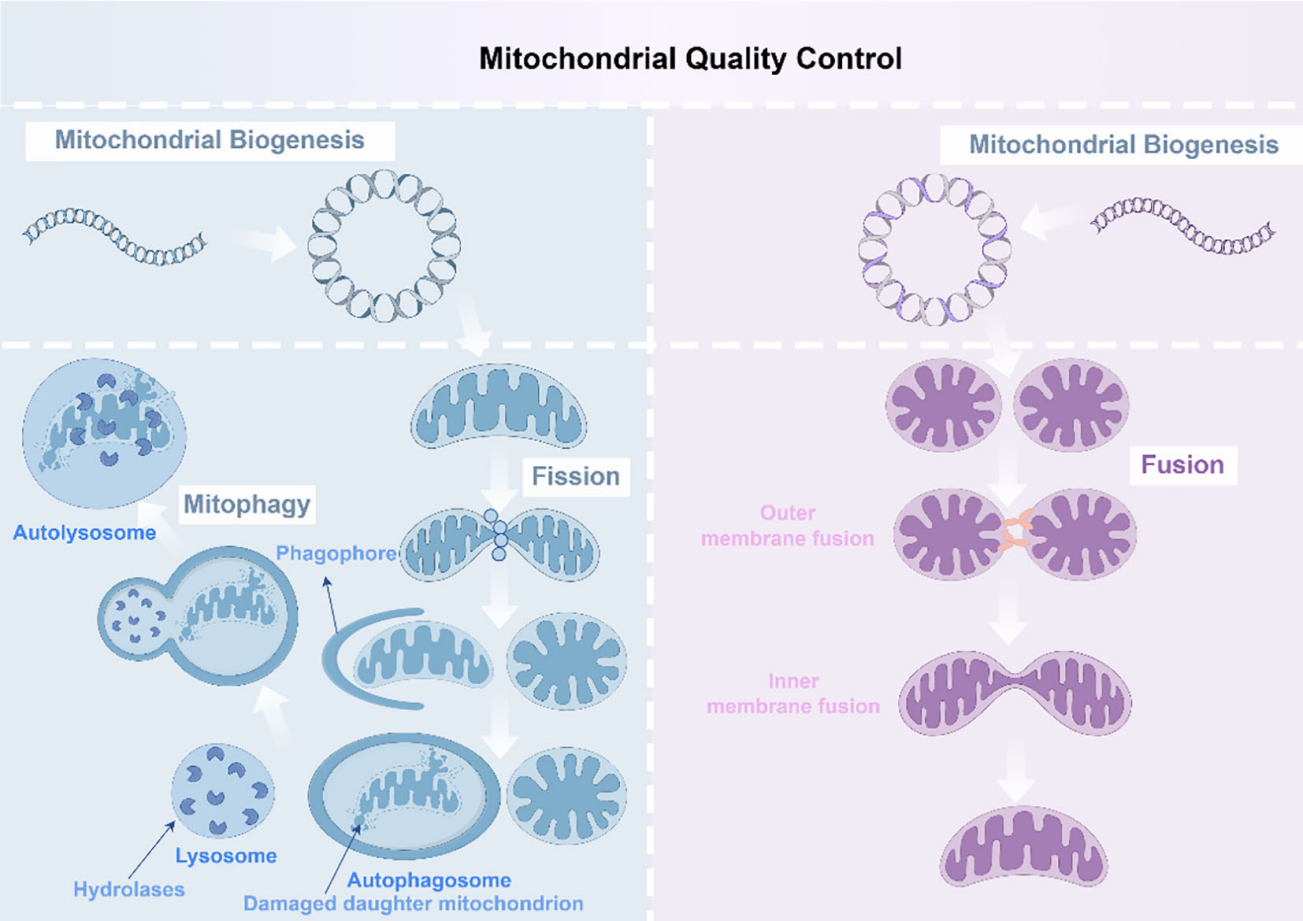
Mitochondrial quality control mechanisms. The mitochondria biogenesis process involves the generation of new mitochondria, through mitochondrial division and the mediation of mitophagy (shown in blue); Mitochondrial biogenesis involves the generation of new mitochondria and the fusion of two mitochondria to form a new one (shown in purple) (By Figdraw).

MQC constitutes an integrated network responsible for monitoring and preserving mitochondrial quality and homeostasis, which are crucial for sustaining mitochondrial health and function. MQC dynamically adjusts its components to meet cellular demands, restoring internal equilibrium during periods of energy depletion or following mitochondrial injury. Mitochondrial morphology is strictly regulated by mitochondrial dynamics and mitophagy. These mechanisms facilitate rapid fusion, exchange of matrix metabolites, and targeted degradation and recycling of irreversibly damaged mitochondria via mitophagy. This prevents damaged or dysfunctional mitochondria from circulating in the healthy mitochondrial pool, thereby maintaining mitochondrial integrity. Dysregulated fission and fusion can impair normal mitochondrial functions. Thus, the coordination between mitochondrial dynamics and mitophagy drives the overall MQC process (20) (**Figure 3**). Furthermore, MQC plays a critical role in myriad physiological and pathological processes, including cell metabolism, differentiation, and oncogenesis.

### 3.1 Mitochondrial biogenesis

Cells initiate mitochondrial biogenesis in response to developmental cues and environmental stressors that alter their energy demands (21). Mitochondrial biogenesis is a process by which new mitochondria are produced from pre-existing ones, involving the coordinated expression of nuclear DNA (nDNA) and mtDNA. Whereas nDNA encodes most proteins essential for mitochondrial structure and function, mtDNA encodes 13 subunits of the respiratory chain that are central to cellular respiration and ATP production (22).

Transcription of mtDNA is orchestrated by transcriptional co-activators encoded by nDNA, notably the peroxisome proliferator-activated receptor γ co-activator 1 (PGC-1) protein family (PGC-1α, PGC-1β, and PGC-1), with PGC-1α being the principal regulator of mitochondrial biogenesis, cell metabolism, and energy supply (23). PGC-1α forms complexes with various transcription factors, including nuclear respiratory factor 1/2 (NRF1/2), estrogen-related receptor alpha (ERRα), and peroxisome proliferator-activated receptor-α (PPARα), through phosphorylation or deacetylation, enhancing transcriptional activity and leading to activation of mitochondrial transcription factor A (TFAM) (24). Proteins encoded by mtDNA are translated with the assistance of nDNA-encoded translation factors, including initiation factors 2 and 3 (mtIF2/3), elongation factor Tu (mtEFTu), elongation factor Ts (mtEFTs), elongation factors G1 (mtEFG1), translational release factor 1-like (mtRF1L), and recycling factors 1 and 2 (mtRRF1 and mtRRF2) (25). Decreased cellular energy levels activate PGC-1α via adenosine monophosphate-activated protein kinase (AMPK) and Sirtuin 1 (SIRT1), upregulating genes associated with mitochondrial biogenesis, increasing the number of mitochondria, and enhancing their function (26, 27). The Hippo signaling pathway also contributes to the regulation of mitochondrial biogenesis. Downstream transcriptional co-activators of the Hippo signaling pathway, namely, Yes-associated protein 1/2 (YAP1/2) and transcriptional co-activator with PDZ-binding motif (TAZ), act as core effectors, influencing cell growth, proliferation, and differentiation by binding to TEA-domain transcription factor 1–4 (TEAD1–4). In endothelial cells, YAP1–TEAD1 complexes regulate PGC1α activity (28). TEAD1 knockdown downregulates PGC1α expression, suppressing mitochondrial biogenesis, glycolysis, and oxygen consumption. Moreover, the S127A mutation of YAP1 limits its phosphorylation and enhances YAP1–TEAD1 binding, resulting in increased PGC1α expression and promoting mitochondrial biogenesis. This suggests that the YAP1–TEAD1 pathway can induce mitochondrial biogenesis via PGC1α in endothelial cells (29).

**Figure 4:**
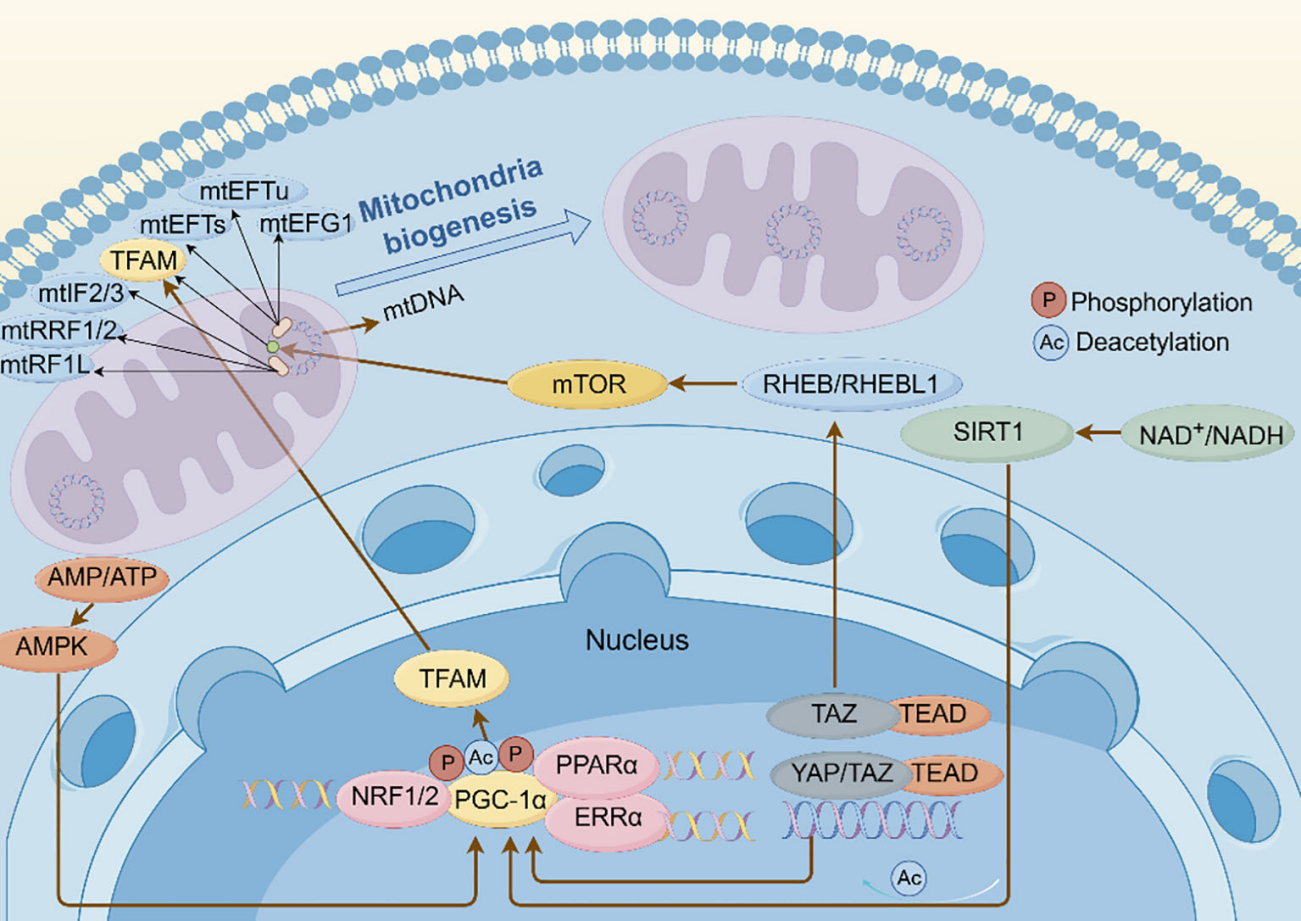
Schematic illustration of the molecular mechanisms mediating mitochondrial biogenesis. When energy demand rises, the master regulator PGC-1α activates transcription of genes encoding mitochondrial building blocks. These components are imported into pre-existing mitochondria, the mitochondrial DNA is replicated, and the organelles enlarge and divide, yielding additional energy-producing mitochondria. AMP, adenosine monophosphate; AMPK, adenosine monophosphate-activated protein kinase; ATP, adenosine triphosphate; EFG1, elongation factor G1; EFTs, elongation factor Ts; EFTu, elongation factor Tu; ERR, estrogen-related receptor; mt, mitochondrial; mTOR, mammalian target of rapamycin; NAD^+^/NADH, nicotinamide adenine dinucleotide; NRF, nuclear respiratory factor; PGC-1α, peroxisome proliferator-activated receptor γ co-activator 1α; PPAR, peroxisome proliferator-activated receptor; Rheb, Ras homolog enriched in brain; SIRT1, Sirtuin 1; TAZ, transcriptional co-activator with PDZ-binding motif; TEAD, TEA-domain transcription factor; TFAM, mitochondrial transcription factor A; YAP, Yes-associated protein (By Figdraw).

TAZ also promotes mitochondrial biogenesis in skeletal muscle through TFAM, mediated by the Ras homolog enriched in brain (RHEB)/RHEB-like 1 (RHEBL1)–mammalian target of rapamycin (mTOR) axis, with TAZ stimulating RHEBl1 expression through TEAD transcription factors (30) (**Figure 4**). Overall, mitochondrial homeostasis depends on the interplay between mitochondrial biogenesis and the removal of damaged mitochondria via mitophagy.

### 3.2 Mitochondrial dynamics

Mitochondria adapt to various cellular environments by dynamically modifying their tubular networks in response to fluctuations in cellular energy requirements. Mitochondrial dynamics involve the strict regulation of continuous fusion and fission processes to maintain mitochondrial homeostasis and respond to energy transfer and cellular signaling. These processes involve remodeling of the membrane structure, which requires interactions between phospholipids and specific proteins. Cardiolipin (CL) and phosphatidic acid (PA) are key phospholipids involved in regulating mitochondrial dynamics to maintain mitochondrial morphology (31). PA is produced in the ER, transported to the OMM, and a portion is converted into CL through a multistep process, contributing to the primary lipid composition of the IMM (32).

### 3.3 Fusion

Mitochondrial fusion is an evolutionarily conserved process that facilitates the exchange of mitochondrial constituents, thereby supporting mtDNA maintenance, mitochondrial respiration, membrane potential equilibrium, apoptosis, and intracellular signaling. In mammals, this process is primarily mediated by three large GTPases associated with mitochondrial dynamics: mitofusin 1 (MFN1), mitofusin 2 (MFN2), and optic atrophy protein 1 (OPA1). Owing to the double-membrane architecture of mitochondria, fusion events occur in two stages: OMM and IMM fusion (33).

MFN1/2 regulate OMM fusion and comprise an N-terminal GTP domain, two C-terminal coiled-coil domains (i.e., heptad repeat 1 [HR1] and 2 [HR2]), and a bipartite carboxy-terminal transmembrane domain. The transmembrane domain anchors MFN1/2 to the OMM, whereas the GTPase and HR1 domains are cytoplasmic, and HR2 extends to the IMS. Homodimerization or heterodimerization of MFN1 and MFN2 on adjacent mitochondria, facilitated by the oligomerization of the HR2 region, brings mitochondrial surfaces together to mediate OMM fusion (34).

OPA1 mediates IMM fusion and maintains cristae structure. Several proteases, including presenilin-associated rhomboid-like protein (PARL), matrix AAA proteases (m-AAA; paraplegin, AFG3L1, and AFG3L2), and IMS AAA proteases (i-AAA; YME1L), process mammalian OPA1, generating the long (L-OPA1) and short (S-OPA1) subtypes. This process is regulated by ATP concentration and IMM potential, demonstrating the regulatory role of the mitochondrial energetic state in OPA1 processing (35). L-OPA1, anchored within the IMM, promotes IMM fusion, whereas S-OPA1 is soluble within the IMS. Fusion efficiency is influenced by the interplay between these two isoforms. Specifically, S-OPA1 can dimerize with L-OPA1, with one region interacting with the target membrane, whereas L-OPA1 binds CL on the target membrane via GTP-independent tethering and facilitates GTP hydrolysis-dependent membrane fusion. The presence of S-OPA1 serves as a regulatory factor in this process (36).

### 3.4 Fission

Mitochondrial fission is crucial for the equitable distribution of mitochondria during cell growth and apoptosis, particularly under conditions of impaired MMP, which is common during the S, G2, and M phases of the cell cycle (37). This process ensures that daughter cells receive an adequate supply of mitochondria. Fission facilitates mitochondrial movement, supports mtDNA replication and cell division, and enables the segregation of damaged mitochondria for subsequent mitophagy. Complementing mitochondrial fusion, fission is a tightly regulated, dynamic process that results in the division of a single mitochondrion into two or more smaller mitochondria. OMM proteins, including mitochondrial fission protein 1 (FIS1), mitochondrial fission factor (MFF), mitochondrial dynamics (MID) protein 51, and MID49, serve as adaptors that recruit mitochondrial dynamin-related protein 1 (DRP1) to sites of division, thus facilitating the removal of damaged segments from the mitochondrial network and establishing the prerequisite conditions for mitophagy (38).

DRP1 is a key regulator of mitochondrial fission and plays a central role in governing mitochondrial dynamics. Upon activation by various signaling pathways, DRP1 translocates from the cytoplasm to the OMM, forming a ring-like polymer at prospective division sites (39). Through GTP hydrolysis, DRP1 induces the constriction and scission of mitochondria in a GTPase-dependent manner, resulting in the formation of daughter mitochondria. The activity and translocation of DRP1 are modulated by post-translational modifications, particularly phosphorylation at S616 and S637, which are also targeted for fission regulation by various viral mechanisms. Additionally, ubiquitination, class-ubiquitination, and sulfhydryl-nitrosylation play roles in regulating the function of DRP1 (40).

**Figure 5:**
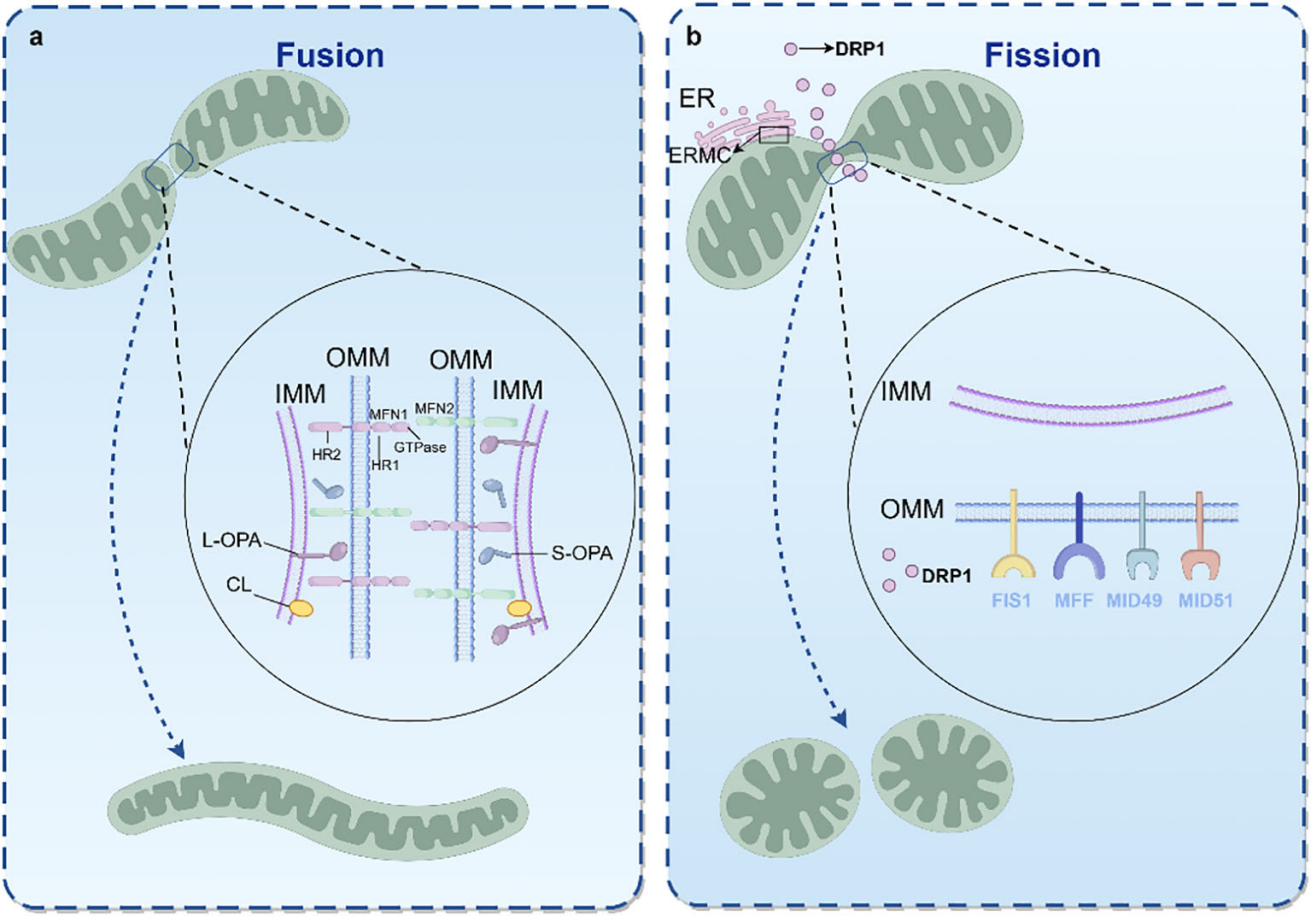
Mitochondrial dynamic mechanisms. **(A)** Mitochondrial fusion process; **(B)** Mitochondrial fission process. DRP1, dynamin-related protein 1; HR, heptad repeat; IMM, inner mitochondrial membrane; MFN, mitofusin; OMM, outer mitochondrial membrane; OPA, optic atrophy protein (By Figdraw).

The ER participates in mitochondrial fission by encapsulating and contracting mitochondria via its tubular architecture, recruiting DRP1 through ER–mitochondria contact sites (ERMC), thereby facilitating recognition and division of mitochondria (41). This ER–mitochondria interaction further stimulates the MAVS-dependent cytoplasmic RNA sensor RIG-I pathway, mediating innate immune responses (42) (**Figure 5**).

### 3.5 Mitophagy

The MQC mechanism regulates damaged mitochondria to maintain homeostasis. Mitophagy selectively eliminates dysfunctional mitochondria, ensuring normal energy production and metabolism. This highly conserved process prevents the accumulation of damaged mitochondria, which would otherwise lead to high oxidative stress levels, reduced ATP levels, and cell injury. Mitophagy is triggered by various stimuli, including nutrient deprivation, hypoxia, excessive ROS accumulation and pathogen invasion (43). It is crucial for cell renewal and repair, energy metabolism, apoptosis, innate immunity and inflammation. The MMP in damaged mitochondria decreases, inducing depolarization. Subsequently, mitochondria are enclosed within autophagosomes and degraded via fusion with lysosomes, a process primarily involving mammalian autophagy-related protein 8 (ATG8) proteins, such as microtubule-associated protein 1 light chain (LC3)A–C and gamma-aminobutyric acid receptor-associated proteins (GABARAP)1–3 (44). Mitophagy operates through ubiquitin-dependent and non-ubiquitin-dependent pathways, depending on the degree of E3 ligase involvement.

### 3.6 Ubiquitin-dependent mitophagy pathway

In mammals, mitophagy primarily clears damaged mitochondria via PTEN-induced putative kinase 1 (PINK1) and E3 ubiquitin ligase Parkin (45). *PINK1* encodes a 581-amino acid protein with a mitochondrial targeting sequence (MTS) at the N-terminus, an outer membrane localization signal, and a transmembrane domain (TMD) (46). Cytoplasmic PINK1 is in its precursor form and acts as a receptor for mitophagy (47). Under normal physiological conditions, cytoplasmic PINK1 localizes to the OMM via MMP. Translocase of OMM 20 (TOMM20), along with TOMM22, recognizes the MTS sequence of PINK1 and guides it through the TOMM40 channel. The positively charged MTS enables PINK1 to cross the IMM with assistance from the TIM23 complex, after which mitochondrial processing peptidases cleave the MTS at amino acid 34, yielding an approximately 60 kDa peptide (48). The subsequent hydrophobic TMD halts further translocation, and PINK1/PGAM5-related PARL protease cleaves within the TMD between A103 and F104, producing an approximately 52 kDa cytoplasmic (c)-PINK1 fragment released into the cytoplasm for degradation by the ubiquitin-proteasome system (49).

cPINK1 overexpression enhances the phosphorylation of AKT S473, thereby activating downstream signaling pathways. Additionally, cPINK1 can directly stimulate the mTOR2 complex, leading to inhibition of mitophagy (50). When mitochondria are damaged, the loss of MMP impairs the PINK1 transport channel, resulting in blocked translocation and subsequent aggregation of PINK1 dimers with OMM proteins (51). Subsequently, PINK1 dimers undergo autophosphorylation, activating the downstream effector Parkin (46). Parkin comprises an N-terminal ubiquitin-like (UBL), C-terminal ring finger domain 1 (RING1), in-between-RING (IBR), and RING2, collectively referred to as RING-between-RING (RBR) domains, and a RING finger-like zinc-binding domain 0 (RING0). As a member of the inter-RING domain families of ubiquitin ligases, Parkin mediates the formation of two polyubiquitin chains and attaches them to OMM proteins via L63 and L27 (52). Ubiquitination is initiated through binding of an E2 enzyme to RING1, followed by transfer of ubiquitin to Parkin’s catalytic C431, forming a homologous to E6AP C-terminus (HECT)-like thioester intermediate, which subsequently leads to substrate ubiquitination (53).

The UBL domain of Parkin also participates in substrate recognition, proteasome binding, and regulating Parkin levels and activities. Structural and biochemical studies have shown that inactive Parkin adopts a self-inhibitory conformation: the UBL domain blocks E2 binding and trans-sulphuration, whereas RING0 prevents C431 from enabling ubiquitination in RING2 (54). PINK1 activates Parkin by phosphorylating its ubiquitin, UBL domains, and neural precursor cell expressed developmentally downregulated protein 8 (NEDD8). NEDD8 is a powerful Parkin binder and activator (55). Once at the OMM, Parkin drives the ubiquitination and proteasomal degradation of several OMM proteins, notably targeting VDAC1 for L27 poly-ubiquitination and mitophagy (52). PINK1 recruits Parkin to the OMM and phosphorylates it at S65, altering its conformation, replacing the UBL, and disrupting the “self-inhibition” state, thus allowing PINK1 to phosphorylate more S65 residues and further stabilizing Parkin’s active conformation (56). Once activated, Parkin attaches ubiquitin chains to the OMM proteins, which then attract autophagy receptors, such as P62/SQSTM1 (sequestosome-1), nuclear dot protein 52 (NDP52), optineurin (OPTN), TAX1 binding protein 1 (TAX1BP1), and neighbor of BRCA1 gene protein (NBR1). These receptors connect the ubiquitinated OMM protein to the autophagosome through the ubiquitin-binding domain (UBD) at one end and LC3 or GABARAP through the LC3-interacting region (LIR) at the other end, facilitating mitophagy by promoting the encapsulation of damaged mitochondria by double-membrane autophagosomes and subsequent fusion with lysosomes (57).

Mitophagy receptors can also recruit autophagy initiation factors, such as UNC-51-like kinase 1 (ULK1), double FYVE-containing protein 1 (DFCP1), and WD repeat domain, phosphoinositide interacting 1 (WIPI1), to promote mitophagy (58). TRAF family member-associated NF-κB activator (TANK) binding kinase 1 (TBK1), 5-azacytidine induced 2 (AZI2, also known as NAP1), and similar to NAP1 and TBK1 adaptor (SINTBAD), outcompete OPTN for TBK1 binding, thereby suppressing OPTN-driven mitophagy. Concomitantly, the released TBK1–AZI2/SINTBAD complexes are redirected to NDP52, where they stabilize the NDP52–focal adhesion kinase family-interacting protein of 200 kDa (FIP200) interaction and potentiate NDP52-mediated mitophagy (59). Ras-related protein Rab-7A (RAB7A) is phosphorylated by TBK1 at S72, further mediating PINK1–Parkin-dependent mitophagy (60). Additionally, MFN2 acts as a scaffold for Parkin translocation upon phosphorylation by PINK1, whereas its degradation may disrupt the ERMC, highlighting the ERMC as a potential key site where PINK1–Parkin activation and deubiquitination events occur (61).

The ubiquitin-dependent mitophagy pathway operates through several E3 ubiquitin ligases other than Parkin. For example, glycoprotein 78 (GP78), an ER membrane-anchored E3 ubiquitin ligase localized in the mitochondrial-associated ER domain, is polyubiquitinated under normal physiological conditions by the cytoplasmic E3 ubiquitin ligase mahogunin RING finger 1 (MGRN1) (62). This process promotes GP78 degradation, thereby maintaining low cellular levels. However, under conditions of cellular stress, such as carbonyl cyanide m-chlorophenyl hydrazone (CCCP) exposure or elevated cytoplasmic Ca^2+^ concentration, GP78 accumulates in the mitochondrial-associated ER domain and mediates mitophagy. Although this process is dependent on GP78, ATG5, and MFN1, it does not require Parkin (63).

Additional pathways involving PINK1, synphilin-1, and seven in absentia homolog 1 (SIAH1) have been reported. Synphilin-1, which binds full-length and cleaved forms of PINK1, is recruited to mitochondria with the assistance of PINK1, leading to mitochondrial depolarization, stabilization of the full-length PINK1 on the OMM, and persistent localization of synphilin-1 on the mitochondria. Subsequently, synphilin-1 binds to the E3 ubiquitin ligase SIAH1, facilitating the polyubiquitination of OMM proteins via the synphilin-1–SIAH1 pathway, and the recruitment of LC3 and lysosomal marker LAMP1 for mitophagy (64).

The mitophagy receptor P62 can also induce mitophagy by increasing mitochondrial superoxide production or anchoring the Kelch-like ECH-associated protein 1 (KEAP1)–E3 ubiquitin ligase RING box protein 1 (RBX1) complex to mitochondria. HB229, an inhibitor targeting the interaction between KEAP1 and NRF2, activates the NRF2 pathway by preventing their association, which induces nuclear translocation of NRF2 and upregulates p62 expression and its accumulation in mitochondria. After ubiquitination of OMM proteins, autophagosomes are recruited into mitochondria through autophagy receptors and LC3, thereby promoting mitophagy (65).

**Figure 6:**
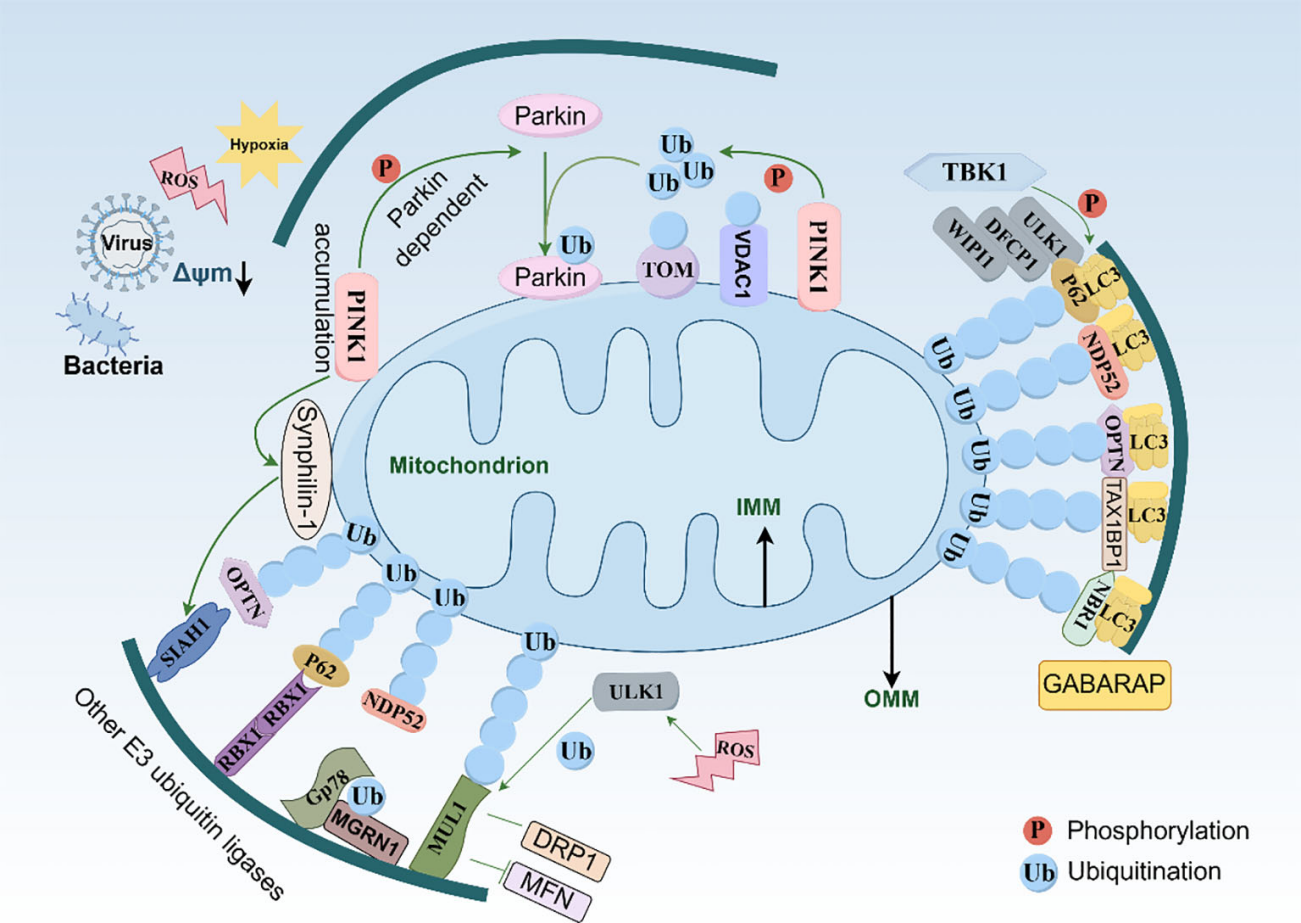
Ubiquitin-dependent mitophagy processes. DRP1, dynamin-related protein 1; GABARAP, gamma-aminobutyric acid receptor-associated protein; IMM, inner mitochondrial membrane; LC3, microtubule-associated protein 1 light chain; MFN, mitofusin; NBRI; NDP52, nuclear dot protein 52; OMM, outer mitochondrial membrane; OPTN; PINK1, PTEN-induced putative kinase 1; ROS, reactive oxygen species; TBK1, TANK-binding kinase 1; TOM, translocase of outer mitochondrial membrane; Ub, ubiquitin; ULK1, UNC-51-like kinase 1; VDAC1, voltage-dependent anion channel protein 1; WIPI1, WD repeat domain, phosphoinositide interacting 1 (By Figdraw).

The mitochondrial E3 ubiquitin ligase 1 (MUL1) induces Parkin-independent ubiquitin-dependent mitophagy through various mechanisms, primarily by stabilizing DRP1 or degrading MFN to maintain mitochondrial homeostasis and facilitate fission (66). Under physiological conditions, MUL1 monitors OMM quality and inhibits ULK1-mediated mitophagy. However, during cellular stress, increased ROS stimulate ULK1 translocation to the mitochondria, initiating mitophagy. Li et al. (67) reported that, following selenite treatment, a fraction of ULK1 translocates to the mitochondria and interacts with MUL1. MUL1 subsequently ubiquitinates ULK1, leading to its degradation, thereby identifying ULK1 as a novel MUL1 substrate (**Figure 6**). Furthermore, Igarashi et al. (68) discovered that the anticancer drug gemcitabine induces mitophagy via MUL1-mediated stabilization of PINK1 on the mitochondrial membrane, independent of mitochondrial depolarization. Multiple additional mitochondrial E3 ubiquitin ligases, including membrane-associated ring finger 5 (MARCH5) and RNF185, have been implicated (69, 70). However, further research is required to elucidate the independent role of Parkin in mediating mitophagy.

### 3.7 Ubiquitin-independent mitophagy pathway

Ubiquitin-independent receptor-mediated mitophagy involves OMM-anchored receptors with (W/F/Y) XX (L/I/V) type LIR motifs that directly bind LC3 or GABARAP to recruit mitochondria to autophagosomes (71). The first mitophagy receptor, ATG32, was identified in yeast, followed by the discovery of five additional receptors in mammalian mitochondria. B-cell lymphoma 2 (BCL2) family proteins play a crucial role in regulating apoptosis and play a significant role in mitophagy. Pro-apoptotic BCL2 members, BCL2/adenovirus E1B 19kD interacting protein (BNIP)3, BNIP 3-like (BNIP3L), and BCL2-like protein 13 (BCL2L13), mediate ubiquitin-independent mitophagy (57, 58), whereas anti-apoptotic FK506 binding protein 8 (FKBP8) acts as a mitophagy receptor that recruits LC3 to damaged mitochondria in an LIR-dependent manner (72). Additionally, the OMM protein FUN14 domain-containing protein 1 (FUNDC1) facilitates hypoxia-induced mitophagy (73).

BNIP3 and BNIP3L/NIX are single BH3 domain BCL2 proteins located in the OMM, with approximately 56% sequence similarity. Both possess conserved structures, atypical BH3 and C-terminal transmembrane (TM) domains, an N-terminal LIR motif, and a presumed short linear motif (SLiM). BNIP3L/NIX has a longer N-terminal sequence but shares a region rich in proline [P], glutamic acid [E], serine [S], and threonine [T] (PEST) sequence in the N-terminal region, suggesting rapid degradation (74). These two proteins dimerize through their C-terminals, anchor to the OMM with their TM and SLiM domains, and use LIR to recruit LC3 or GABARAP and induce mitophagy (44). Phosphorylation at specific serine residues enhances their interaction with LC3 or GABARAP (BNIP3: LIR S17/S24; BNIP3L/NIX: LIR S34/S35, S81). BNIP3 preferentially binds to LC3 (74, 75), whereas BNIP3L/NIX preferentially binds to GABARAP (76). Formation of the BNIP3L/NIX–LC3 complex enhances the recruitment of mitochondrial autophagosomes (77).

BCL2L13 promotes apoptosis via a mitochondrial-dependent pathway and serves as a mitophagy receptor in mammals. Unlike BNIP3 and BNIP3L/NIX, BCL2L13 contains four BH domains, two LIR motifs, and a BHNo region with tandem repeats, as well as a common TM motif anchoring it to the OMM (78). Mutations in the BH domains reduce the ability of BCL2L13 to induce mitophagy (79). Although mouse BCL2L13 features two N-terminal WXXL/I motifs, only mutation of the second (LIR2) impairs mitophagy induction, identifying LIR2 as the binding site for LC3 or GABARAP (78). Phosphorylation of S272 in BCL2L13 promotes LC3 binding, analogous to S114 in ATG32 (79). BCL2L13 also promotes mitochondrial fission and can induce fragmentation and mitophagy independent of DRP1 and Parkin, provided that it does not have mutations in its BH1–4 and TM domains (75).

Similar to BCL2L13, FKBP8 is a mammalian homolog of yeast ATG32 identified as a mitophagy receptor within the immunophilin protein family (80). It is unique among the FKBP family members for possessing a TM structure and is primarily localized to the OMM. The N-terminal region of FKBP8 contains the LIR sequence (FEVL), a peptidyl-prolyl cis/trans isomerase (PPIase) domain, three tetratricopeptide repeat (TPR) motifs, and a Ca^2+^-calmodulin binding region. Its PPIase activity is induced by Ca^2+^-calmodulin and exhibits pronounced anti-apoptotic properties by recruiting BCL2/Bcl-XL to the mitochondria and LC3A via the LIR motif. Moreover, FKBP8 interacts strongly with LC3A, LC3B, GABARAP, and GABARAPL1 (72).

Yoo et al. (81) identified a conserved LIR motif-like sequence (LIRL), located approximately 70 residues from the canonical LIR motif, which is preserved between multiple species. This sequence is closely related to mitochondrial fragmentation and operates in conjunction with LIR through interactions with LC3, thereby participating in mitophagy. Notably, FKBP8-induced mitochondrial fragmentation is independent of DRP1, BNIP3, and BNIP3L/NIX, but requires OPA1 (80). FKBP8 typically mediates mitophagy under nutrient deprivation, with its absence abolishing starvation-induced autophagy activation, whereas its overexpression promotes the autophagic cascade. The initiation of autophagosome formation is regulated by the phosphatidylinositol 3-kinase VPS34 complex, wherein ATG14L co-localizes with BECN1 and FKBP8; FKBP8 is essential for modulating VPS34 activity (82). Distinct from other mitophagy receptors, FKBP8 avoids lysosomal degradation during mitophagy by translocating from the mitochondria to the ER. Although the precise mechanism underlying this process is unclear, it may play a critical role in sustaining mitophagy and inhibiting apoptotic signaling throughout the process (83).

**Figure 7:**
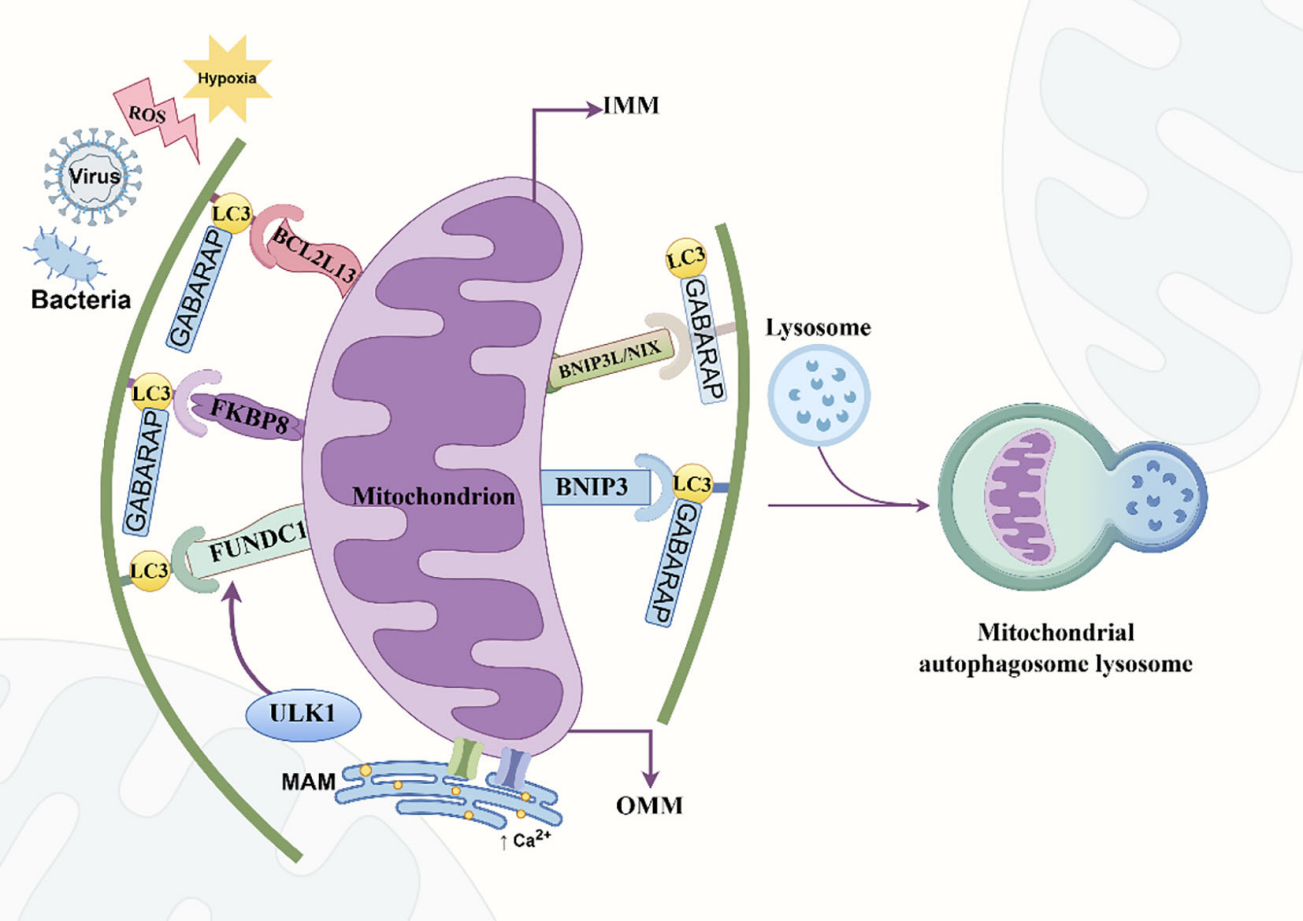
Ubiquitin-independent mitophagy processes. BNIP, BCL2/adenovirus E1B 19kD interacting protein; FKBP8, FK506 binding protein 8; FUNDC1, FUN14 domain-containing protein 1; GABARAP, gamma-aminobutyric acid receptor-associated protein; IMM, inner mitochondrial membrane; LC3: microtubule-associated protein 1 light chain; MAM, mitochondria-associated membrane; OMM, outer mitochondrial membrane; ROS, reactive oxygen species; ULK1, UNC-51-like kinase 1 (By Figdraw).

FUNDC1 is a novel mitophagy-related receptor that facilitates cellular adaptation to hypoxia by regulating mitophagy. The cytoplasmic N-terminal region of FUNDC1 contains the LIR motif (YXXL) that binds LC3 to initiate mitophagy. The TM domain at the C-terminus anchors FUNDC1 to the OMM (71). Mutations or deletions in the LIR region affect FUNDC1-mediated mitophagy through three key phosphorylation sites: Y18, S13, and S17. Dephosphorylation of Y18 and S13, and phosphorylation of S17, enhances FUNDC1 interaction with LC3 and promotes autophagosome recruitment (84). Specifically, casein kinase 2 and SRC protein tyrosine kinase phosphorylate Y18 site and S13, respectively, suppressing FUNDC1–LC3B binding. The mitochondrial phosphatase phosphoglycerate mutase family member 5 (PGAM5) dephosphorylates S13, promoting the interaction between FUNDC1 and LC3B (85). Additionally, the anti-apoptotic protein BCL2L1 binds to PGAM5 and inhibits its phosphatase activity, thereby promoting S13 phosphorylation and inhibiting FUNDC1-induced mitophagy (86). Additionally, ULK1, a key autophagy-initiating kinase, phosphorylates S17, promoting the interaction between FUNDC1 and LC3, a critical step for FUNDC1-mediated mitophagy (**Figure 7**) (87).

Under hypoxic conditions, FUNDC1-driven mitophagy requires interactions between the ER and mitochondria. Specifically, FUNDC1 binds to the ER-resident protein calnexin within the mitochondria-associated membrane (MAM), facilitating mitochondrial division, ULK1 recruitment, and subsequent phosphorylation of S17, thereby promoting mitophagy under hypoxic conditions (88). Although FUNDC1-mediated mitophagy operates independently of Parkin, FUNDC1 can also be ubiquitinated, functioning as a novel substrate for the mitochondrial E3 ubiquitin ligase, MARCH5. Chen et al. (89) demonstrated that MARCH5 mediates the ubiquitination of FUNDC1 at K119, and the subsequent degradation of FUNDC1 impairs hypoxia-induced mitophagy. In contrast, endogenous FUNDC1 enhances this process.

Tang et al. (90) observed that MARCH5 inhibits mitophagy-mediated cell pyroptosis by degrading FUNDC1, affecting cell proliferation, migration, and invasion, and contributing to thyroid cancer progression. In endometrial cancer, increased FUNDC1 expression correlates strongly with immune markers and enhances chemosensitivity to carboplatin and paclitaxel, demonstrating its potential value as a prognostic biomarker and therapeutic target (91). Despite these advancements, current knowledge regarding how post-translational modifications such as phosphorylation and dephosphorylation affect FUNDC1-mediated mitophagy is limited, and further research is required.

## 4 Role of MQC in viral infections

### 4.1 Effects of viruses on mitochondrial biogenesis

Viruses dynamically regulate mitochondrial biogenesis by targeting and modulating key molecular pathways to accommodate their replication requirements. Certain viruses suppress mitochondrial biogenesis to facilitate their replication, whereas others enhance it to support host cell survival and enable sustained viral replication. Different viruses use different mechanisms to affect mitochondrial generation. Typically, viral interference with mitochondrial biogenesis leads to alterations in mitochondrial functionality, thereby supporting viral translation and assembly processes (92).

A central regulatory pathway of mitochondrial biogenesis is the PGC-1α–NRF1–TFAM axis. Aberrant expression within this pathway has been documented in diseases caused by RNA and DNA viruses, demonstrating a link between viral infection and mitochondrial biogenesis (**Table 1**). For example, RNA viruses, such as the hepatitis C virus (HCV), induce DNA damage in T cells and contribute to immune system dysfunction. Liquid chromatography-mass spectrometry analyses revealed increased mtROS production and decreased mtDNA content, coupled with downregulated PGC-1α and TFAM expression. These findings suggest that disrupting PGC-1α and TFAM may lead to mitochondrial impairment and impair mitochondrial biogenesis during chronic HCV infection (93). However, in the context of HCV infection and hepatic gluconeogenesis, PGC-1α, which is strongly induced under starvation conditions, acts as a transcriptional coactivator that initiates gluconeogenic pathways and participates in energy metabolism. Oxidative stress, as reflected by elevated mtROS during HCV infection, can further increase hepatic PGC-1α expression, promoting gluconeogenesis and inhibiting insulin signaling, potentially leading to insulin resistance and diabetes. The differential regulatory effects of HCV on PGC-1α may be cell model-dependent (94).

**Table 1:** The role of mitochondrial biogenesis in viral infection.

Virus type	Virus name	Virus abbreviation	Target	Mechanism	Outcome	Infection stage
RNA Virus	Hepatitis C Virus	HCV	PGC-1α, TFAM	Downregulates PGC-1α and TFAM expression; induces DNA damage in T cells	Impaired mitochondrial biogenesis; decreased mtDNA content; increased mtROS production	Chronic Infection
RNA Virus	Hepatitis C Virus	HCV	PGC-1α	Upregulates PGC-1α via oxidative stress (mtROS)	Enhanced gluconeogenesis; inhibited insulin signaling; potential insulin resistance and diabetes	Chronic Infection
DNA Virus	Hepatitis B Virus	HBV	PGC-1α	Utilizes PGC-1α to enhance viral transcription (especially after cytotoxic anticancer drug treatment)	Promoted HBV replication	Chronic Infection
DNA Virus	Bovine Herpesvirus Type 1	BoHV-1	NRF1/2, TFAM	Stimulates TFAM expression independently of NRF1/2 pathway	Excessive ROS production; mitochondrial damage; aberrant expression of respiratory chain proteins and antioxidant enzymes	Acute Infection
DNA Virus	Epstein-Barr Virus	EBV	NRF1, TFAM	Downregulates NRF1 and TFAM expression	Reduced mitochondrial number; inhibited mitochondrial biogenesis	Lytic Infection
DNA Virus	Epstein-Barr Virus	EBV	PGC-1α	LMP1 increases PGC-1α methylation via PRMT1 interaction	Enhanced PGC-1α stability	Latent Infection
DNA Virus	Herpes Simplex Virus 1	HSV-1	NRF1, TFAM	TBK1 phosphorylates NRF1 at S318, inactivating NRF1-TFAM axis	Decreased TFAM activity; increased mtDNA release; enhanced antiviral response	Lytic Infection

Among DNA viruses, hepatitis B virus (HBV) uses PGC-1α to increase HBV transcription, particularly following treatment with cytotoxic anticancer drugs, thereby mediating HBV replication (95). In advanced bovine herpesvirus type 1 (BoHV-1) infection, host cells experience excessive ROS production and pronounced mitochondrial damage, including aberrant expression of respiratory chain proteins and antioxidant enzymes, as well as NRF1/2 and TFAM. BoHV-1 infection stimulates TFAM expression independently of NRF1/2, although the precise mechanism remains unclear (96). Epstein-Barr virus (EBV) infection reduces the number of mitochondria by downregulating the expression of NRF1 and TFAM, inhibiting mitochondrial biogenesis (97). The latent membrane protein 1 (LMP1) of EBV, an oncogenic factor, increases PGC-1α methylation through interactions involving protein arginine methyltransferase 1 (PRMT1) and PGC-1α, thereby enhancing PGC-1α stability (98). However, further research is required to investigate the broader effects of EBV infection on mitochondrial biogenesis.

Key regulators of mitochondrial biogenesis also influence innate antiviral immunity. In mice, inhibiting NRF1 impairs mitochondrial biogenesis, increases virus-induced mitochondrial damage, promotes the release of mtDNA and mtROS, and activates the innate immune response through damage-associated molecular patterns (DAMPs). During herpes simplex virus 1 (HSV-1) infection, TBK1 phosphorylates NRF1 at S318, thereby inactivating the NRF1–TFAM axis. Disruption of this pathway increases TFAM activity, reduces mtDNA release, and weakens the HSV-1-induced antiviral response (24). Thus, mitochondrial biogenesis is crucial for innate antiviral defense, although its precise role warrants further investigation.

Collectively, these studies demonstrate that the PGC-1α–NRF1–TFAM axis does not operate as a tightly interconnected cascade; rather, each component can function independently in distinct viral infection models.

### 4.2 Effects of viruses on mitochondrial dynamics

Viral infections induce various physiological and biochemical changes in host cells, directly affecting mitochondrial homeostasis and activating MQC to regulate mitochondrial quantity, morphology, and networking (**Table 2**). Mitochondrial dynamics rely on a balance of fission and fusion for normal function, with excessive fission triggering mitophagy. TBK1 phosphorylates S637 on DRP1 upon sensing viral RNA or DNA signals, thereby inhibiting its GTPase activity and preventing its aggregation on the OMM. This inhibits mitochondrial fission, promotes mitochondrial fusion, enhances MAVS signaling, amplifies IFN expression, and strengthens the antiviral response (99). Viruses develop strategies to counteract these mechanisms, often exploiting mitochondrial dynamics for replication (3).

**Table 2:** Mechanisms of mitochondrial dynamics in viral infection.

Virus type	Virus name	Virus abbreviation	Viral protein / factor	Target	Mechanism	Outcome	Infection stage
RNA Virus	Zika Virus	ZIKV	NS4A	–	Drives mitochondrial fission	Inhibits MAVS-mediated IFN response	Acute Infection
RNA Virus	Zika Virus	ZIKV	NS2B3, NS3	MAVS	Downregulates MAVS expression; NS3 binds 14-3-3 motif of MAVS	Inhibits IFN release; Prevents RIG-I/MDA5 translocation to mitochondria	Acute Infection
RNA Virus	Dengue Virus	DENV	NS4B, NS3	DRP1	Promotes fusion and elongation by reducing DRP1 S616 phosphorylation	Supports replication; Weakens RIG-I-dependent innate immune response	Acute Infection
RNA Virus	Dengue Virus	DENV	NS2B3 (Protease)	MFN1, MFN2	Cleaves MFN1/2 to inhibit mitochondrial fusion	MFN1: disrupts antiviral RLR signaling. MFN2: disrupts MMP maintenance; Promotes infection	Acute Infection
RNA Virus	Hepatitis C Virus	HCV	NS5A	MMP, ROS	Increases ROS, disrupts MMP, promotes fission, recruits Parkin	Triggers fission; Mediates mitophagy; Supports replication	Acute Infection
RNA Virus	Hepatitis C Virus	HCV	(General)	DRP1	Induces DRP1 phosphorylation and translocation to mitochondria	Suppresses fusion; Supports viral replication	Acute Infection
RNA Virus	Bovine Viral Diarrhea Virus	BVDV (NCP)	Erns (LIR motif)	DRP1, Parkin	Triggers DRP1 translocation and S616 phosphorylation (fission); Erns acts as a mitophagy receptor to induce Parkin-mediated mitophagy	Inhibits MAVS- and mtDNA-cGAS-mediated innate immunity; Blocks apoptosis and inflammatory response	Acute Infection
RNA Virus	SARS-CoV	SARS-CoV	ORF-9b	DRP1, MAVS	ORF-9b localizes to mitochondria, triggers ubiquitination and proteasomal degradation of DRP1 (causing elongation); Usurps PCBP2 and AIP4 to degrade MAVS/TRAF3/TRAF6	Causes mitochondrial elongation; Decreases IFN production; Blocks RIG-I–MAVS signaling; Induces autophagy	Acute Infection
RNA Virus	Nervous Necrosis Virus	NNV	(General)	DRP1, MFF	Promotes DRP1 Ser616 phosphorylation and translocation (MFF-dependent) to induce mitochondrial fission	Weakens RLR signaling and MAVS-mediated downstream signaling	Acute Infection
DNA Virus	Kaposi’s Sarcoma-associated Herpesvirus	KSHV	vBCL-2	NM23-H2, DRP1	Binds NM23-H2 to stimulate GTP loading of DRP1	Causes mitochondrial fission; Inhibits MAVS aggregation; Impairs IFN response	Lytic Replication
DNA Virus	Epstein-Barr Virus	EBV	LMP2A	Notch Pathway, DRP1	Induces DRP1 upregulation via Notch signaling	Enhances mitochondrial fission; Promotes cell migration and invasion (EMT)	Latent Infection
DNA Virus	Epstein-Barr Virus	EBV	BHRF1	DRP1, MAVS	Targets mitochondria promoting DRP1-mediated fission	Inhibits MAVS-mediated IRF3 and IFN-I innate immune responses	Lytic Infection (Reactivation)

Virus-induced disruption of MMP releases mtDNA and mtROS, altering mitochondrial dynamics. Many viruses or their proteins induce mitochondrial fission directly or indirectly by upregulating fission proteins or downregulating fusion proteins, disrupting MAVS signaling, inhibiting innate immunity, delaying apoptosis to favor replication, and later promoting apoptosis to release new viral particles, effectively manipulating mitochondria to serve viral needs, including the provision of energy required for assembling new viral particles (100). In particular, MAVS is central to the immune response against RNA viruses. MFF facilitates DRP1 recruitment and MAVS cluster formation on mitochondria. Under conditions of mitochondrial dysfunction, AMPK phosphorylation of MFF disrupts MAVS clustering and weakens acute antiviral responses (101). MFF can also be selectively spliced, regulating various mitochondrial functions including energy metabolism and cell differentiation (102).

RNA viruses often promote mitochondrial fission and suppress fusion, impairing MAVS signaling and diminishing RIG-I/MDA5–MAVS-mediated IFN response (103). Members of the *Flaviviridae* family, including Zika virus (ZIKV) and dengue virus (DENV), are key examples (11). ZIKV non-structural protein 4A (NS4A) drives mitochondrial fission and inhibits MAVS-mediated IFN responses (104), whereas NS2B3 and NS3 downregulate MAVS expression and inhibit IFN (especially IFNβ) release. Additionally, ZIKV NS3 inhibits RLR signaling by binding to the 14-3–3 binding motif of MAVS, thereby preventing the translocation of RIG-I and MDA5 to mitochondria (105).

DENV affects mitochondrial morphology differently depending on the viral strain and associated structural proteins. NS4B and NS3 promote mitochondrial fusion and elongation, which support DENV replication and weaken RIG-I-dependent innate immune responses by reducing DRP1 S616 phosphorylation (106). Conversely, NS2B3 impairs mitochondrial fusion, with the fusion proteins MFN1 and MFN2 playing different roles in this process. MFN1 mediates the antiviral RLR pathway to inhibit DENV replication, whereas MFN2 helps maintain MMP to alleviate DENV-induced cell death. DENV protease cleaves MFN1/2 to inhibit mitochondrial fusion, promoting DENV infection by disrupting IFN production and MMP (107). Thus, different DENV non-structural proteins regulate mitochondrial dynamics in distinct ways.

HCV induces DRP1 phosphorylation and its translocation to mitochondria, triggering fission and suppressing fusion, which supports viral replication (100). HCV NS5A increases ROS, disrupts MMP, promotes mitochondrial fission, and recruits Parkin to mediate mitophagy (108).

Non-cytopathic (NCP) bovine viral diarrhea virus (BVDV) of the *Pestivirus* genus recruits DRP1 to mitochondria, promoting S616 phosphorylation and mitochondrial fission. This triggers Parkin-mediated mitophagy to clear damaged mitochondria and suppresses MAVS and mtDNA–cGAS-mediated innate immune responses. Released mtROS induces apoptosis and inflammation, regulating NCP BVDV replication (109).

Severe acute respiratory syndrome coronavirus 2 (SARS-CoV-2) uses its open reading frame 9b (ORF-9b) to target mitochondria by ubiquitinating DRP1, causing mitochondrial elongation and promoting proteasomal degradation. ORF-9b also binds MAVS, blocking RIG-I–MAVS signaling and promoting K48-linked ubiquitin-dependent proteasomal MAVS degradation, which decreases IFN production (110). SARS-CoV-2 infection lowers circulating cell-free mitochondrial DNA (ccf-mtDNA) levels. Thus, ccf-mtDNA levels are a useful marker of mitochondrial dysfunction due to incomplete mitophagy in SARS-CoV-2 infection (111).

Kaposi’s sarcoma-associated herpesvirus (KSHV) uses BCL-2 (vBCL-2) to bind the host nucleoside diphosphate (NDP) kinase NM23-H2, inducing GTP loading of DRP1 GTPase, causing mitochondrial fission, inhibiting MAVS aggregation, and impairing the IFN response (112). Similarly, in fish, nervous necrosis virus (NNV) of the *Nodaviridae* family disrupts mitochondrial dynamics by promoting DRP1 and MFF-dependent mitochondrial fission, weakening RLR signaling and MAVS-mediated downstream signaling to evade the innate immune response (113).

DNA viruses, such as EBV, induce DRP1 upregulation through the Notch signaling pathway via LMP2A, leading to enhanced mitochondrial fission in gastric and breast cancer cells (114). Similar to RNA viruses, EBV-encoded BCL2 homolog 1 (BHRF1) disrupts mitochondrial dynamics by targeting mitochondria and promoting DRP1-mediated mitochondrial fission, inhibiting MAVS-mediated IRF3 and IFN-I innate immune responses (115).

Taken together, these findings highlight the key role of mitochondrial dynamics in viral replication and host innate immunity.

### 4.3 Effects of viruses on ubiquitin-dependent mitophagy

Viruses disrupt mitochondrial function to enhance their replication and propagation within host cells. Although mild mitochondrial damage leads to recycling via mitochondrial dynamics, severe damage triggers mitophagy, which has a dual role: It supports host immune defenses by regulating innate immune responses and apoptosis, but it can also be exploited by viruses to evade immunity and facilitate their replication (16) (**Table 3**).

**Table 3:** Mechanisms of ubiquitin-dependent mitophagy in viral infection.

Virus type	Virus name	Abbreviation	Viral protein	Target	Mechanism	Outcome	Infection stage
DNA Virus	Herpes Simplex Virus 1	HSV-1	(General)	ICP34.5, US11; EIF2S1–ATF4 axis, Parkin	Regulates EIF2S1–ATF4 to inhibit Parkin expression; Blocks Parkin-dependent mitophagy	Accumulation of damaged mitochondria; Supports viral replication and survival	Acute Infection
DNA Virus	Herpes Simplex Virus 1	HSV-1	(General)	VMP1, WHSC1L1	Downregulates VMP1 (promotes mitophagy); Upregulates WHSC1L1 to inhibit VMP1 via histone modification	Inhibits mitophagy; Supports viral replication	Acute Infection
RNA Virus	Porcine Epidemic Diarrhea Virus	PEDV	Structural protein N	PINK1–Parkin, MFN2, JAK1–STAT1	Initiates mitophagy via MFN2 ubiquitination through PINK1–Parkin pathway; Interacts with TRIM28	Suppresses apoptosis; Dampens innate immune responses (JAK1–STAT1); Promotes replication	Acute Infection
DNA Virus	Varicella-Zoster Virus	VZV	Glycoprotein E (GE)	LC3, PINK1–Parkin, DRP1, MAVS, STING	Interacts with LC3 to upregulate mtROS and promote PINK1–Parkin-dependent mitophagy; Inhibits MAVS oligomerization and STING translocation	Interferes with MAVS- and STING-mediated IFN responses; Promotes replication	Acute Infection
RNA Virus	Foot-and-Mouth Disease Virus	FMDV	(General)	HSP60, DRP1, Parkin	Induces mitophagy early in infection via HSP60 depletion leading to DRP1 phosphorylation and Parkin recruitment	Suppresses its own replication (early stage)	Acute Infection
RNA Virus	Enterovirus 71	EV71	(General)	PINK1–Parkin, MAVS	Initiates PINK1–Parkin-dependent mitophagy; Promotes MAVS degradation/cleavage	Dampens innate immunity; Boosts viral replication	Acute Infection
DNA Virus	Pseudorabies Virus	PRV	(General)	PINK1–Parkin, MAVS	Mediates PINK1–Parkin-dependent mitophagy; Degrades MAVS on the OMM	Reduces IFN-I production; Promotes viral replication	Acute Infection
DNA/RNA Virus	Singapore Grouper Iridovirus / Red-spotted Grouper NNV	SGIV / RGNNV	(General)	Parkin, IFN-β, NF-κB	Upregulates Parkin expression; Suppresses IFN-β and NF-κB promoter activity	Suppresses innate immune response; Facilitates viral replication	Acute Infection

Disruption of MMP or increased oxidative stress levels can activate PINK1–Parkin signaling pathway-mediated ubiquitin-dependent mitophagy. Early in infection, mitophagy helps contain viral replication by removing infected or damaged mitochondria. Later, viruses may evade or exploit mitophagy to support their replication, depending on the virus type, strain, and cell type (116). HSV-1 infection causes damaged mitochondria to accumulate in neurons and mouse brain tissues by first activating, then inhibiting mitophagy. HSV-1 dysregulates the eukaryotic translation initiation factor 2 subunit 1 (EIF2S1)–activating transcription factor 4 (ATF4) via infected cell protein 34.5 (ICP34.5) or unique short protein 11 (US11), inhibiting Parkin expression and blocking Parkin-dependent mitophagy. Although inhibiting mitophagy with MDIVI-1 promotes HSV-1 infection, overexpression of Parkin or administration of mitophagy activators such as CCCP, rotenone, or taurine inhibits infection and reduces NF-κB-mediated inflammation (117). Epigenetically, HSV-1 downregulates vacuole membrane protein 1 (VMP1), which promotes mitophagy, and upregulates histone methyltransferase Wolf-Hirschhorn syndrome candidate 1-like 1 (WHSC1L1), which inhibits VMP1 via histone modification and DNMT3A recruitment. However, these effects can be reversed by an autophagy inhibitor (3-MA) and downregulation of WHSC1L1. These results suggest that HSV-1 manipulates these pathways to support its replication and survival (118).

Porcine epidemic diarrhea virus (PEDV) infection causes mitochondrial damage and triggers mitophagy in African green monkey kidney cells (Vero) and porcine intestinal epithelial cells (IPEC-J2), promoting viral replication. PEDV structural protein N initiates mitophagy via MFN2 ubiquitination through the PINK1–Parkin signaling pathway, thereby suppressing apoptosis, inhibiting the JAK1–STAT1 pathway, dampening innate immune responses, and promoting PEDV replication (119). TRIM28 also induces mitophagy by interacting with PEDV N protein through its RING domain. CCCP treatment reduces the effect of TRIM28 on JAK–STAT1 activation and pSTAT nuclear translocation (120).

Varicella-zoster virus (VZV) triggers DRP1-mediated mitochondrial fission during infection and subsequently activates PINK1–Parkin-dependent mitophagy, promoting viral replication. VZV glycoprotein E (GE) upregulates mtROS by interacting with LC3 to promote PINK1–Parkin-dependent mitophagy. GE also inhibits MAVS oligomerization and STING translocation, interfering with MAVS- and STING-mediated IFN responses. This suppression of IFN responses is associated with PINK1–Parkin-dependent mitophagy. In a three-dimensional human skin organ culture model, CCCP-mediated mitophagy enhanced VZV replication and decreased IFN production (121).

Foot-and-mouth disease virus (FMDV) induces mitophagy at the early stage of infection to suppress its own replication, a process coordinated by heat shock protein 60 (HSP60). HSP60 depletion leads to DRP1 phosphorylation at S616 and translocation, inducing Parkin-mediated mitophagy (122). Enterovirus 71 (EV71) infection similarly causes mitochondrial damage and initiates PINK1–Parkin-dependent mitophagy, suppressing MAVS-driven immune responses. Silencing PINK1 inhibits EV71 replication and MAVS cleavage, whereas overexpressing Parkin has the opposite effect. Inhibiting mitophagy with cyclosporin A (CsA) lessens EV71-mediated pathological damage and virus replication, indicating that EV71 exploits PINK1-mediated mitophagy to dampen innate immunity and boost viral replication (123).

Pseudorabies virus (PRV) infection disrupts mitochondrial structure and function, leading to MMP depolarization, fewer mitochondria, and imbalanced dynamics. PRV mediates PINK1–Parkin-dependent mitophagy to phagocytize damaged mitochondria and reduce IFN-1 production. Mitophagy leads to the degradation of MAVS on the OMM, promoting viral replication (124).

Grouper spleen (GS) cell lines infected with Singapore grouper iridovirus (SGIV) or red-spotted grouper NNV (RGNNV) exhibit upregulated Parkin expression, which suppresses IFN-β and NF-κB promoter activity, and IFN-related factor and cytokine expression, leading to increased LC3 expression. Thus, Parkin overexpression facilitates the replication of SGIV and RGNNV (125).

### 4.4 Effects of viruses on ubiquitin-independent mitophagy

Ubiquitin-independent receptor-dependent mitophagy is also important in viral infection disease models (**Table 4**). Mitophagy receptors with LIR domains bind directly to LC3 or GABARAP to induce mitophagy, typically under hypoxic conditions or during ROS accumulation (126). Ubiquitin-dependent and ubiquitin-independent mitophagy can function separately, together, or alternately, thereby creating a multi-layered defense network. In coxsackievirus B3 (CVB3) infection, which causes viral myocarditis (VMC), Parkin and BNIP3-mediated mitophagy are triggered, whereas autophagic flux is blocked by inhibiting autophagosome–lysosome fusion. Silencing Parkin decreases mitophagy activity, causing damaged mitochondria to accumulate and exacerbating VMC-induced apoptosis (127).

**Table 4:** Mechanisms of receptor-mediated ubiquitin-independent mitophagy in viral infection.

Virus type	Virus name	Abbreviation	Viral protein	Target	Mechanism	Outcome	Infection stage
RNA Virus	Coxsackievirus B3	CVB3	(General)	Parkin, BNIP3	Triggers Parkin- and BNIP3-mediated mitophagy but blocks autophagosome–lysosome fusion	Silencing Parkin decreases mitophagy, causing damaged mitochondria accumulation and exacerbating apoptosis	Acute infection
RNA Virus	Influenza Virus	IFV	NS1	ULK1, BNIP3	Induces mitochondrial damage/fission; Upregulates expression of ULK1 and BNIP3	Mediates mitophagy	Acute infection
RNA Virus	Southern Rice Black-Streaked Dwarf Virus	SRBSDV	NSP7-1	BNIP3, ATG8, AMPKα	Interacts with BNIP3 and ATG8; Promotes BNIP3 dimerization; Stimulates AMPK phosphorylation (AMPKα–BNIP3 pathway)	Induces receptor-dependent mitophagy	Persistent infection
RNA Virus	Bovine Parainfluenza Virus Type 3 / Vesicular Stomatitis Virus	BPIV3 / VSV	(General)	ENTR1, NIX, MAVS	ENTR1 inhibits NIX-dependent mitophagy; Stabilizes MAVS; Enhances type I interferon (IFN-I) response	Inhibits viral replication	Acute infection

Influenza virus (IFV) NS1 induces mitochondrial damage, increases mitochondrial fission, and upregulates the expression of ULK1 and BNIP3, thereby mediating mitophagy (128). Similarly, the fiber structure formed by the NSP7–1 of southern rice black-streaked dwarf virus (SRBSDV) interacts with BNIP3 and targets mitochondria to induce mitophagy. SRBSDV or NSP7–1 alone can promote BNIP3 dimerization on mitochondria and induce mitophagy via interaction with ATG8. Additionally, SRBSDV stimulates AMPK phosphorylation, which subsequently promotes BNIP3 phosphorylation via the AMPKα–BNIP3 pathway, further facilitating mitophagy (129).

Receptor-mediated mitophagy also plays a critical role in host innate immune function; for example, Yao et al. reported that endosome-associated trafficking regulator 1 (ENTR1) stabilizes mitochondrial antiviral-signaling protein (MAVS) by inhibiting NIX-dependent mitophagy, thereby enhancing the type I interferon (IFN-I) response and inhibiting the replication of bovine parainfluenza virus type 3 (BPIV3) and vesicular stomatitis virus (VSV) (130).

### 4.5 Effects of viruses on mitophagy receptors

Mitophagy receptors, such as P62, NDP52, and OPTN, are crucial in virus-mediated mitophagy, recruiting mitophagy-related proteins and binding to LC3 and ubiquitinated substrates to remove damaged mitochondria. For example, HCV NS5A recruits selective autophagy receptors NDP52 and OPTN and autophagosome-related proteins (ATG14 and ULK1, ATG5, and DFCP1) to mitochondria, initiating mitophagy via the PINK1–Parkin pathway (131). However, these receptors can aid in clearing viruses and can be exploited for viral replication (**Table 5**).

**Table 5:** Mechanisms of mitophagy receptor in viral infection.

Virus type	Virus name	Abbreviation	Viral protein	Target	Mechanism	Outcome	Infection stage
RNA Virus	Hepatitis C Virus	HCV	NS5A	NDP52, OPTN, PINK1-Parkin	Recruits selective autophagy receptors (NDP52, OPTN) and autophagosome-related proteins to mitochondria	Initiates mitophagy via the PINK1–Parkin pathway	Persistent Infection
RNA Virus	Coronaviruses	CoV	(General)	P62, PDPK1	PDPK1 phosphorylates P62 at T138, shifting virophagy to mitophagy	Helps viruses evade the innate immune response	Acute Infection
RNA Virus	Infectious Bursal Disease Virus	IBDV	RNA polymerase VP1	OPTN	Interacts directly with OPTN	Interferes with polymerase activity; Inhibits viral replication	Acute Infection
DNA Virus	African Swine Fever Virus	ASFV	P17	TOMM70, P62, MAVS	Binds TOMM70 to promote P62-TOMM70 interaction, inducing mitophagy and MAVS degradation	Decreases mitochondria number; Inhibits production of IFN-α, IL-6, and TNF-α; Regulates innate immune response	Acute Infection

Coronaviruses induce virophagy and mitophagy; P62 promotes the degradation of viral M protein and mitochondria, whereas phosphoinositide-dependent kinase-1 (PDPK1) is a negative regulator of innate immunity, shifting virophagy to mitophagy by phosphorylating the T138 of P62, helping viruses evade the innate immune response. Targeting PDPK1 can restore innate immunity and suppress RNA virus replication (132).

The RNA polymerase viral protein 1 of infectious bursal disease virus (IBDV) interacts directly with OPTN, which interferes with its polymerase activity, thereby inhibiting viral RNA synthesis, and activating antiviral innate immunity via IFN signaling to inhibit IBDV replication (133).

Viral proteins also regulate P62 indirectly. For example, the African swine fever virus (ASFV) P17 binds TOMM70, which promotes binding between P62 and TOMM70, inducing mitophagy and decreasing the number of mitochondria. TOMM20 and TOMM70 expression levels decrease significantly following the expression of P17 or ASFV infection. Moreover, P17-mediated mitophagy leads to MAVS degradation and inhibits the production of IFN-α, IL-6, and TNF-α, thereby regulating the innate immune response (134).

### 4.6 Mitochondrial Tu translation elongation factor acts as a “bridge” for mitophagy in viral infection

Mitochondrial Tu translation elongation factor (TUFM) is central to mitochondrial protein synthesis, mitophagy regulation, antiviral immunity, and cellular metabolism (135). Several viruses exploit TUFM to promote replication. The severe fever with thrombocytopenia syndrome virus (SFTSV) nucleoprotein (NP) interacts with TUFM, facilitating its translocation to mitochondria and mediating mitochondrial isolation in autophagosomes via LC3, which induces mitophagy and degrades MAVS signals, thereby evading host immune responses and promoting viral replication (136). The NS1 protein of respiratory syncytial virus (RSV) uses an LIR motif to bind LC3 and TUFM, acting as a mitophagy receptor that suppresses RIG-1 and IFN responses, thereby supporting RSV replication (137). The influenza A virus (IAV) non-structural protein polymerase basic protein 1-frame 2 (PB1-F2) interacts with TUFM and translocates to mitochondria, mediating mitochondrial damage, inducing fission and mitophagy via LC3B interactions, and degrading MAVS to inhibit IFN-1 release and suppress innate immunity. The C-terminal LIR motif is key for these effects (138). This aligns with the mechanism whereby the HPIV3 matrix protein (M) mediates mitophagy and inhibits type I interferon (IFN-I) secretion (139). Additionally, the H1N1 IAV NP mediates mitophagy and MAVS degradation through a crucial Y313 residue, blocking MAVS-mediated antiviral signaling and aiding viral replication by interacting with MAVS and the autophagy receptor Toll-interacting protein (TOLLIP) (140).

### 4.7 Relationship between mitophagy and apoptosis in viral infection

Apoptosis, a programmed cell death process, is another antiviral defense mechanism. Viral infection activates the caspase cascade to degrade viral components and promotes the formation of apoptotic bodies, which are phagocytosed by neighboring immune cells to inhibit viral replication and release (141). Viruses adapt by manipulating apoptosis and mitophagy. Early mitophagy aids viruses by engulfing damaged mitochondria and preventing the release of ROS and Cyt-C, thereby limiting apoptosis and enabling replication. However, excessive mitophagy can lead to cellular energy depletion and mitochondrial dysfunction, promoting apoptosis (16). For example, Bombyx mori cypovirus (BmCPV), a member of the *Reoviridae* family, encodes a viral small peptide comprising approximately 59 amino acid residues (VSP59), which interacts with prohibitin 2, an IMM autophagy receptor, triggering caspase 3-dependent apoptosis and reducing viral replication. This demonstrates the role of small viral peptides in host–virus interactions associated with mitophagy and apoptosis (142).

The non-structural protein Pns11 of the arthropod-borne rice gall dwarf virus (RGDV) hijacks the fiber structure formed by VDAC1, causing mitochondrial degeneration. Subsequently, damaged mitochondria are engulfed by the PINK1–Parkin pathway. VDAC1 regulates apoptosis by controlling the release of apoptotic signaling molecules through its pores. Gelsolin (GSN), an anti-apoptotic protein, binds VDAC1. The interaction between Pns11, GSN, and VDAC1 reduces VDAC1 expression and increases GSN, thereby blocking apoptosis in virus-infected sites. Mitophagy also suppresses apoptotic responses (143).

In summary, viruses mediate mitophagy through multiple pathways, transforming their protective function for the cell into a means for viral replication. This suggests that targeting mitophagy has potential as a mechanism for blocking chronic viral infection.

## 5 Antiviral pharmacological targeting of MQC

Viruses exploit host endogenous pathways and subcellular structures, especially mitochondria, to support their replication by altering mitochondrial function and cell metabolism. Many viruses manipulate MQC mechanisms, such as biogenesis, dynamics, and mitophagy, and induce mitochondrial metabolic reprogramming to meet their needs for viral replication. Understanding how MQC is regulated during antiviral responses and identifying antiviral drug targets within these processes is particularly important.

### 5.1 Targeting mitochondrial biogenesis

Viruses obtain energy and raw materials via mitochondrial biogenesis. Antiviral therapies targeting mitochondrial biogenesis function by inhibiting the proliferation and functional remodeling of the mitochondria, limiting the energy and biosynthetic resources required for viral propagation (**Table 6**). These therapies may also regulate the PGC-1α-NRF1/2–TFAM axis to target mitochondrial biogenesis, providing a plausible antiviral strategy (24). PGC-1α serves as a central regulator of energy metabolism, orchestrating essential processes, such as mitochondrial biogenesis, gluconeogenesis, and FA oxidation through complex molecular mechanisms. Curcumin, a polyphenolic compound derived from turmeric, exhibits anti-inflammatory, antioxidant, and antitumor activities. PGC-1α participates in the coordinated transcription and replication of HBV, whereas curcumin inhibits HBV replication by downregulating PGC-1α expression, possibly through direct proteolytic degradation. Thus, targeting PGC-1α is a promising approach for suppressing HBV replication (144).

**Table 6:** Mechanisms of antiviral drugs targeting mitochondrial biogenesis.

Agent / compound	Source / type	Target	Target virus	Mechanism	Outcome	Regulatory stage(s) of viral infection
Sulforaphane (SFN)	Natural isothiocyanate (Cruciferous vegetables)	NRF2	HIV-1	Activates NRF2; Blocks nuclear import of viral pre-integration complex	Inhibits HIV-1 infection in macrophages	Post-entry, Pre-integration (Specifically Nuclear Import Stage)
Isoliquiritigenin (ISL)	Natural chalcone (Licorice roots)	NRF2	VSV, H1N1, EMCV, HSV-1	Acts as an NRF2 agonist; Independent of interferon pathway	Inhibits viral replication	Post-Entry Stage
Ginsenoside Rg5	Ginseng extract	NRF2	HSV-1	Induces NRF2 expression; Reverses HSV-1-induced ROS and NF-κB activation	Inhibits HSV-1 replication; Neuroprotective	Replication Stage
Lanatoside C (LanC)	Natural cardiac glycoside (Digitalis lanata)	NRF2	HSV-1	Mediates NRF2 perinuclear translocation (ring-like structure around nucleus)	Inhibits HSV-1 replication	Replication Stage
Bardoxolone methyl (CDDO-Me)	Synthetic triterpenoid (Derivative of oleanolic acid)	NRF2	RABV	Activates NRF2 and downstream cytoprotective genes (HO-1, NQO1)	Inhibits RABV infection	Replication Stage
Curcumin	Polyphenolic compound (Turmeric)	PGC-1α	HBV	Downregulates PGC-1α expression (promotes degradation)	Inhibits HBV replication	Transcription Stage
Resveratrol	Natural polyphenol (Grapes, berries)	SIRT1–NRF2–HO-1 pathway	RABV	Activates antioxidant pathway; Directly inactivates virus particles; Inhibits multiple life cycle steps	Reduces virus-induced oxidative stress; Inhibits RABV infection	Adsorption, Replication, Release, Viral Inactivation
MOTS-c	Mitochondrial-derived short peptide	MAVS / MYH9-actin complex	HBV	Enhances mitochondrial biogenesis and MAVS signaling pathway; Regulates mitochondrial dynamics	Suppresses HBV replication; Improves hepatic function	Replication Stage (via Mitochondrial Dynamics)

Transcription factors NRF1 and NRF2 interact synergistically with PGC-1α to regulate mitochondrial gene expression and biogenesis and are components of redox homeostasis (145). Isoliquiritigenin (ISL), a natural chalcone compound extracted from licorice roots, acts as an NRF2 agonist and demonstrates antiviral activity against HCV and IAV. ISL effectively inhibits the replication of vesicular stomatitis virus, H1N1, encephalomyocarditis virus, and HSV-1. Notably, the absence of NRF2 diminishes this antiviral effect (146). HIV-1 infection also significantly downregulates NRF2 in host cells and activates ROS-mediated signaling via NF-κB signaling, thereby promoting viral replication. Ginsenoside Rg5 (G-Rg5), extracted from ginseng, induces NRF2 nuclear translocation and upregulation, thereby reversing the HSV-1-induced increase in ROS and NF-κB activation, and inhibiting HIV-1 replication. Thus, G-Rg5 holds potential as a targeted NRF2 modulator for anti-HSV-1 therapy (147).

Sulforaphane (SFN), a natural isothiocyanate found in cruciferous vegetables like broccoli and kale, exerts antioxidant and anti-inflammatory properties by activating NRF2. SFN can directly or indirectly modulate factors related to mitochondrial biogenesis, thereby improving mitochondrial function. It can also effectively inhibit HIV-1 infection in primary macrophages and phorbol 12-myristate 13-acetate-induced monocyte cell lines. Silencing NRF2 enhances HIV-1 replication and decreases the antiviral effect of SFN, suggesting that SFN bolsters antiviral effects by coordinating NRF2-mediated HIV-1 inhibition (148).

Lanatoside C (LanC), an ion transport regulator approved by the United States Food and Drug Administration (FDA), possesses antitumor and antiviral properties. LanC has demonstrated efficacy in inhibiting HSV-1 replication *in vitro* and *in vivo*, with the associated mechanism mediated by NRF2 nuclear translocation. LanC thus has potential for use as a novel NRF2-targeted therapy for HSV-1 infection (149).

Bardoxolonemethyl (CDDO-Me), another FDA-approved agent, is a potent NRF2 agonist. CDDO-Me inhibits different strains (SC16, CVS-11, and CTN) of rabies virus (RABV) infection in the mouse neuroblastoma cell line, Neuro2a. Treatment with all-trans retinoic acid, an NRF2-specific inhibitor, significantly attenuates this effect, indicating that the antiviral effect of CDDO-Me may rely on NRF2 activation (150). Similarly, resveratrol activates the SIRT1–NRF2–HO-1 antioxidant pathway to inhibit RABV infection during viral adsorption, replication, and release, directly inactivating the virus and reducing virus-induced oxidative stress. However, it has no significant effect on the internalization process. This multi-target mechanism demonstrates its therapeutic potential against RABV (151).

Additionally, the mitochondrial-derived short peptide MOTS-c enhances mitochondrial biogenesis and MAVS signaling during HBV infection. MOTS-c suppresses HBV replication and improves hepatic function without significant toxicity, presenting a promising new avenue for HBV treatment (152).

### 5.2 Targeting mitochondrial dynamics

Viruses directly or indirectly impair mitochondrial function, disrupt mitochondrial homeostasis, and use various strategies to interfere with mitochondrial dynamics. In this way, viruses undermine the host cell’s antiviral defense mechanisms to promote their replication. Damaged mitochondria may be reintegrated via mitochondrial dynamics, whereas viruses exploit this mechanism by manipulating mitochondrial fission and fusion to promote persistent infections [1]. The regulation of mitochondrial dynamics varies among viruses and at different stages of infection. Restoring the balance between mitochondrial fission and fusion represents a promising antiviral strategy (**Table 7**).

**Table 7:** Mechanisms of antiviral drugs targeting mitochondrial dynamics.

Agent / compound	Source / type	Target	Target virus	Mechanism	Outcome	Regulatory stage(s) of viral infection
MDIVI-1	Synthetic small molecule inhibitor (Laboratory synthesis)	DRP1	PRV, ECTV	Inhibits DRP1 GTPase activity, reducing mitochondrial fission; Restores mitochondrial function	Inhibits PRV infection (restores MMP, decreases ROS); Attenuates ECTV-mediated inhibition of MAVS immunity	Replication Stage
Icariside II (ICS II)	Natural compound (Epimedium)	FIS1, DRP1	HBV	Upregulates FIS1 expression to promote mitochondrial fission (facilitates DRP1 recruitment)	Reduces ROS; Remodels mitochondrial network; Disrupts metabolic environment for viral replication	Replication Stage
Ginsenoside Rg3 (G-Rg3)	Ginseng extract	CDK1–DRP1 pathway	HCV	Inhibits the CDK1–DRP1 pathway; Corrects abnormal mitochondrial division; Inhibits excessive division-induced mitophagy	Potent inhibitor of HCV replication	Replication Stage
Melatonin	Endogenous hormone	Ca^2+^, ROS, YAP–Hippo–OPA1, DRP1	EV71	Reduces Ca^2+^ accumulation and ROS; Promotes fusion (via YAP–Hippo–OPA1); Inhibits fission; Restores DRP1 expression	Inhibits EV71 replication; Ameliorates severe mitochondrial damage	Replication Stage
VBNI-1	Small molecule	vBCL-2–NM23-H2 complex	KSHV	Targets vBCL-2–NM23-H2 complex; Inhibits excessive mitochondrial division; Enhances MAVS recruitment; Activates TBK1/IRF3	Restores mitochondrial immune signaling; Inhibits KSHV replication and virion production	Late Stage (specifically Virion Morphogenesis, Assembly, and Nuclear Egress)
Mito-C	Small organic molecule (Heterocyclic compound)	NEET protein family (NAF-1)	DENV	Binds to NAF-1 (key regulator of dynamics); Modulates DRP1-induced fragmentation; Reverses virus-induced excessive mitochondrial fusion	Suppresses DENV genome replication and virion assembly	Replication Stage

MDIVI-1, a common compound that modulates mitochondrial dynamics by inhibiting the GTPase activity of DRP1 and reducing mitochondrial fission, demonstrates broad-spectrum antiviral effects. For example, during PRV infection, mitochondrial fusion is downregulated and fission is upregulated; treatment with MDIVI inhibited PRV infection, restored mitochondrial function, increased MMP, and decreased ROS levels (153). Similarly, in ectromelia virus (ECTV) infection, MDIVI-1 alters mitochondrial morphology, attenuates ECTV-mediated inhibition of MAVS-dependent immunity, and reduces viral replication in L929 fibroblasts (154).

Icariside II (ICS II), extracted from Epimedium, has been identified as a potential anti-HBV agent, upregulating FIS1 expression and promoting mitochondrial fission. FIS1 acts as a DRP1 receptor, facilitating its recruitment to the mitochondrial membranes. Additionally, ICS II effectively reduces ROS levels in HBV-infected cells, remodels the mitochondrial network, improves oxidative stress, and disrupts the metabolic environment required for viral replication, thereby exerting anti-HBV effects (155).

Ginsenoside Rg3 (G-Rg3) regulates mitochondrial dynamics, inhibits the cyclin-dependent kinase 1 (CDK1)–DRP1 pathway in HCV infection, corrects abnormal mitochondrial division, inhibits viral replication, and mitigates excessive division-induced mitophagy. Thus, G-Rg3 is a potent inhibitor of HCV replication (156).

Melatonin, an endogenous hormone synthesized and metabolized within mitochondria, contributes to MQC regulation. It primarily maintains mitochondrial dynamic homeostasis by reducing Ca^2+^ accumulation and ROS production, promoting mitochondrial fusion, inhibiting fission, and activating the YAP–Hippo signaling pathway to enhance OPA1-mediated fusion (157). In human neuroblastoma SK-N-SH cells infected with EV71, melatonin restores DRP1 expression, inhibits EV71 replication, and ameliorates severe mitochondrial damage (158).

KSHV vBCL-2 binds to NM23-H2 to promote DRP1-mediated mitochondrial fission, thereby supporting continuous viral replication. The small molecule VBNI-1 targets the vBCL-2–NM23-H2 complex, inhibits excessive mitochondrial division, enhances MAVS recruitment to mitochondria, and activates TBK1 and IRF3 signaling pathways. This restores mitochondrial immune signaling, effectively inhibits KSHV replication, and prevents virion production (112).

The small organic molecule drug Mito-C is a heterocyclic compound that targets the NEET protein family, specifically binding to NAF-1, a key regulator of mitochondrial dynamics. NAF-1 drives DRP1-induced mitochondrial network fragmentation. Mito-C modulates this process and, in the context of DENV infection, reverses the virus-induced excessive mitochondrial fusion, suppressing DENV genome replication and virion assembly. This provides a new targeted strategy for antiviral therapy (41).

### 5.3 Targeting mitophagy

Mitophagy, as a form of selective autophagy, plays a crucial role in maintaining mitochondrial homeostasis within cells. Viral infections often alter the physiological and biochemical functions of host cells, leading to damage in the mitochondrial network and subsequently inducing mitophagy to enhance mitochondrial integrity. Certain viruses have evolved strategies to manipulate mitophagy, using it for their replication and modulating key mitochondrial-localized immune molecules to evade host immune attacks (159). Accordingly, numerous small molecule mitophagy regulators have been identified as potential antivirals, offering valuable insights into mitophagy mechanisms and drug development (**Table 8**).

**Table 8:** Mechanisms of antiviral drugs targeting mitophagy.

Agent / compound	Source / type	Target	Target virus	Mechanism	Outcome	Regulatory stage(s) of viral infection
CCCP	Synthetic chemical uncoupler (Laboratory synthesis)	MMP, ROS	DPV	Induces mitophagy; Reduces ROS production; Inhibits DPV-mediated apoptosis	Inhibits DPV infection (dose-dependent)	Intercellular Transmission (Cell-to-cell spread)
CCCP / Rotenone / Taurine	Synthetic / Natural (Lab synthesis / Plant roots)	Parkin	HSV-1	Restores mitophagy; Upregulates Parkin expression (Taurine)	Suppresses HSV-1 replication; Decreases NF-κB-mediated neuroinflammation	Replication Stage
Matrine	Natural alkaloid (Sophora flavescens)	RLR pathway	DHAV-1	Promotes mitophagy; Mitigates mitochondrial damage and pyroptosis; Suppresses excessive RLR activation	Inhibits viral replication; Alleviates cell damage	Excessive Interferon & Pyroptosis
Baicalin	Natural flavonoid (Scutellaria baicalensis)	IFN-1	NIBV	Inhibits NIBV-triggered mitophagy; Preserves mitochondrial function; Augments IFN-1 production	Enhances innate immune response; Inhibits viral replication	Innate Immunity & Macrophage Polarization
Tanreqing (TRQ)	Traditional Chinese Medicine formulation	mtROS, NLRP3	IAV H1N1	Induces mitophagy to remove dysfunctional mitochondria; Decreases mtROS and NLRP3 activation	Reduces IL-1β release; Disrupts viral replication	NLRP3 Inflammasome Activation
Xijiao Dihuang + Yinqiao (XDY)	Traditional Chinese Medicine formulation	ROS–NLRP3 axis	IFV	Enhances mitophagy factor expression; Inhibits ROS–NLRP3–pyroptosis axis	Improves pulmonary inflammation; Inhibits IFV replication	NLRP3 Inflammasome Activation
Liang–Ge–San (LGS)	Traditional Chinese Medicine formulation	α7nAChR, ROS	SARS-CoV-2, H1N1	Upregulates α7nAChR; Activates cholinergic anti-inflammatory pathway; Inhibits mitophagy; Suppresses ROS	Alleviates lung pathology and inflammation	Acute Lung Injury & Cytokine Storm
Niclosamide	Synthetic antiparasitic agent	MMP, PINK1–Parkin	ZIKV	Disrupts IMM proton gradient; Decreases MMP; Initiates PINK1–Parkin-dependent mitophagy	Removes damaged mitochondria; Suppresses ZIKV replication	Mitochondrial Fragmentation & Viral Replication
ALT001	Synthetic therapeutic candidate (Novel)	ULK1/RAB9, PINK1–Parkin	HSV-1	Restores mitophagy (counteracting US3 inhibition); Repairs mitochondrial damage; Activates IFN immunity	Inhibits HSV-1 infection; Restores Aβ phagocytosis; Reduces neuroinflammation	Microglial Inflammation & Viral Replication
TZOA	Synthetic derivative (Arctigenin derivative)	MAVS	IHNV	Promotes mitochondrial fusion and mitophagy; Restores MAVS-mediated IFN pathway	Enhances innate antiviral response; Reduces mortality in juvenile rainbow trout	Mitochondrial Homeostasis & Innate Immunity

CCCP, a lipophilic weak acid proton shuttle, enhances lipid membrane proton permeability and serves as an effective disruptor of MMP, thereby inducing mitophagy. It inhibits duck plague virus (DPV) infection in duck embryonic fibroblasts in a dose-dependent manner by activating mitophagy, reducing ROS production, and inhibiting DPV-mediated apoptosis, indicating its potential as a candidate antiviral agent against DPV (160).

In HSV-1 infection, mitophagy is activated during early stages but is suppressed later due to decreased Parkin expression, thereby inhibiting mitophagy. Overexpression of Parkin or treatment with mitophagy activators, such as CCCP or rotenone, restores mitophagy, suppresses HSV-1 replication, and decreases NF-κB-mediated neuroinflammation. Additionally, taurine, identified as a differential metabolite following HSV-1 infection *in vivo* and *in vitro*, functions as a mitophagy activator to upregulate Parkin expression, stimulate mitophagy, and inhibit HSV-1 infection (117). Studies focusing on VZV infection have shown that CCCP-induced mitophagy increases viral replication and attenuates the IFN response in three-dimensional human skin organ culture models (121). Previous studies have demonstrated that CCCP exerts inhibitory effects on specific viruses. Further studies are warranted to investigate the breadth of its antiviral activity. These results demonstrate the variable roles of mitophagy in different viral infections.

Natural compound drugs also exert antiviral effects by regulating mitophagy. The plant alkaloid, Matrine, has anti-inflammatory and antiviral effects and promotes mitophagy in duck hepatitis A virus type 1 (DHAV-1) infection, mitigating mitochondrial damage and cell pyroptosis, suppressing excessive RLR pathway activation triggered by mitochondrial damage, reducing downstream production of IFN, and inhibiting viral replication. Hence, Matrine may serve as an effective method for treating DHAV-1 infection and other hepatitis viruses (161).

Baicalin, a natural flavonoid compound, ameliorates mitochondrial damage induced by nephropathogenic infectious bronchitis virus (NIBV) infection, inhibits NIBV-triggered mitophagy, preserves mitochondrial function, augments IFN-1 production to enhance the innate immune response, and inhibits viral replication (162).

Traditional Chinese medicine formulations, such as Tanreqing (TRQ) and Xijiao Dihuang decoction combined with Yinqiao powder (XDY) have demonstrated antiviral efficacy through mitophagy modulation. TRQ induces mitophagy in IAV H1N1-infected mouse mononuclear macrophages J774A.1 to remove dysfunctional mitochondria, decreases mtROS accumulation, and NLRP3 inflammasome activation, thereby reducing pro-inflammatory IL-1β release and disrupting viral replication (163). XDY improves pulmonary inflammation in mice with IFV-induced pneumonia by enhancing mitophagy factor expression, inhibiting the ROS–NLRP3–pyroptosis axis, promoting complete autophagic circulation, and inhibiting IFV replication (164). Liang-Ge-San (LGS) upregulates α7 nicotinic acetylcholine receptor (α7nAChR) expression in viral acute lung injury mediated by SARS-CoV-2 and H1N1, activating the cholinergic anti-inflammatory pathway, inhibiting mitophagy, suppressing ROS production, and alleviating lung pathology and inflammation caused by viral infection (165).

Chemically synthesized agents also regulate mitophagy during viral infections. For example, the mitochondrial uncoupling agent niclosamide, initially approved for use as an antiparasitic agent, exhibits favorable mitophagy regulatory and antiviral effects against ZIKV. Niclosamide disrupts the proton gradient of the IMM, decreasing MMP and initiating PINK1–Parkin-dependent mitophagy to remove damaged mitochondria and suppress ZIKV replication (166).

TZOA, a synthetic arctigenin derivative, alleviates mitochondrial damage, promotes mitochondrial fusion and mitophagy, and restores the IFN pathway mediated by MAVS, enhancing the innate antiviral response and inhibiting viral infection in an *in vitro* Epithelioma papulosum cyprini cell line of infectious hematopoietic necrosis virus (IHNV) infection. In juvenile rainbow trout, TZOA reduces the mortality rate induced by IHNV and significantly prolongs the survival period, and also reduces the transmission efficiency of IHNV. This offers a potentially new approach for controlling aquatic viruses and establishes a theoretical basis for developing antiviral drugs that target the interaction between mitochondria and the immune system (167).

HSV-1 contributes to neurodegenerative disease pathology by promoting amyloid protein β (Aβ) accumulation, mitochondrial damage, and neuroinflammation. The US3 protein kinase of HSV-1 inhibits PINK1–Parkin-mediated mitophagy, leading to mitochondrial dysfunction in human and mouse microglial cells. ALT001, a novel therapeutic candidate for Alzheimer’s disease, restores mitophagy, through ULK1/RAB9 pathway activation, repairs HSV-1-induced mitochondrial damage, activates IFN-mediated antiviral immunity, and inhibits HSV-1 infection. Additionally, ALT001 restores Aβ phagocytosis by microglia and reduces neuroinflammation. Thus, its antiviral–mitochondrial repair–inflammation relief triple mechanism positions ALT002 as a potentially important candidate anti-HSV-1 drug (168).

Currently, most antiviral interventions targeting mitophagy have been validated only *in vitro* and *in vivo*, with no clinical application. Thus, further research is warranted to fully elucidate their therapeutic potential.

## 6 Conclusions

MQC is essential for maintaining mitochondrial homeostasis, especially during viral infections that cause mitochondrial damage. The MQC mechanism, which is targeted by most viruses, affects different stages of the virus life cycle. MQC is vital for host defense; therefore, it is often hijacked by viruses to promote their replication, playing a dual role in viral infections. Viruses alter mitochondrial functions and evade innate immune responses to sequester metabolic intermediates, thereby maintaining viral replication. However, the exact mechanism by which viruses regulate MQC remains unclear. Host cells are sensitive to viral infection-induced perturbations in the microenvironment. Furthermore, the effects of viral proteins are diverse and vary in specificity depending on the stage of infection. Viral-mediated regulation of MQC, whether direct or indirect, is a complex process. Therefore, identifying the mitochondrial targets of viral action is essential to develop therapeutic interventions for viral infectious diseases.

This review outlines how viral proteins manipulate host mitochondria to regulate infection and discusses recent advances in small molecule pharmacology targeting these processes. As viruses and hosts co-evolve and new survival mechanisms are discovered, understanding protein–protein interactions is vital in predicting viral infection outcomes. Host cells are highly influenced by their microenvironment; therefore, future studies conducting mechanistic analyses should consider host-cell specificity, viral protein structural homology, and local stress levels. Additionally, identifying the common signaling pathways and effectors among viruses would facilitate the development of broad-spectrum antiviral therapies. Mitochondria, which play a crucial role in innate immune responses and the regulation of cell death, remain a key area of research focus. Exploring the mechanisms underlying innate immune responses triggered by mitochondria and how they are affected by MQC would provide novel insights into mitochondrial regulation in viral infections and aid in the development of innovative antiviral therapies. Currently, research on the mechanisms of viral-induced mitochondrial metabolic reprogramming remains an emerging field.

## Glossary

ATP: adenosine triphosphate
AMPK: adenosine monophosphate-activated protein kinase
ATF: activating transcription factor
ASFV: African swine fever virus
ATG: autophagy-related protein
BCL2: B-cell lymphoma 2
BCL2L: BCL2-like protein
BoHV-1: bovine herpesvirus type 1
BNIP: BCL2/adenovirus E1B 19kD interacting protein
BVDV: bovine viral diarrhea virus
CCCP: carbonyl cyanide m-chlorophenyl hydrazone
CDDO-Me: bardoxolonemethyl
cGAMP: cyclic guanosine monophosphate-adenosine monophosphate
cGAS: cGAMP synthase
CL: cardiolipin
CsA: cyclosporin A
CSFV: classical swine fever virus
CVB3: Coxsackievirus B3
Cyt-C: cytochrome C
DAMPs: damage-associated molecular patterns
DENV: Dengue virus
DFCP1: double FYVE-containing protein 1
DRP1: dynamin-related protein 1
EBV: Epstein-Barr virus
EIF: eukaryotic translation initiation factor
ER: endoplasmic reticulum
ERMC: endoplasmic reticulum–mitochondria contact site
ERR: estrogen-related receptor
EV71: Enterovirus 71
FIS1: fission protein 1
FKBP: FK506 binding protein
FMDV: foot-and-mouth disease virus
FUNDC: FUN14 domain-containing protein
GP: glycoprotein
G-Rg3: Ginsenoside Rg3
HBeAg: HBV e antigen
HBV: hepatitis B virus
HCV: hepatitis C virus
HECT: homologous to E6AP C-terminus
HPV: human papillomavirus
HR: heptad repeat
HSV: herpes simplex virus 1
IAV: influenza A virus
IFN: interferon
IFV: Influenza virus
IHNV: infectious hematopoietic necrosis virus
IMM: inner mitochondrial membrane
IMS: intermembrane space
IRF: interferon regulatory factor
JAK: Janus kinase
KEAP1: Kelch-like ECH-associated protein 1
KSHV: Kaposi’s Sarcoma-associated herpesvirus
LanC: Lanatoside C
LC3: microtubule-associated protein 1 light chain
LGP2: laboratory of genetics and physiology 2
LGS: Liang-Ge-San
LIR: LC3-interacting region
LMP1: latent membrane protein 1
MAM: mitochondria-associated membrane
MARCH5: membrane-associated ring finger 5
MARV: Marburg virus
MAVS: mitochondrial antiviral signaling protein
MDA5: melanoma differentiation-associated gene 5
MFF: mitochondrial fission factor
MFN: mitofusin
MID: mitochondrial dynamics protein
MMP: mitochondrial membrane potential
MQC: mitochondrial quality control
mtDNA: mitochondrial DNA
mTOR: mammalian target of rapamycin
mtROS: mitochondrial reactive oxygen species
mtDAMP: mitochondrial damage-associated molecular pattern
MTS: mitochondrial targeting sequence
MUL1: mitochondrial E3 ubiquitin ligase 1
NAD: nicotinamide adenine dinucleotide
NADH: nicotinamide adenine dinucleotide
NCP: non-cytopathic
nDNA: nuclear DNA
NDP52: nuclear dot protein 52
NDV: Newcastle disease virus
NF-κB: nuclear factor kappa-light-chain-enhancer of activated B
NLR: NOD-like receptor
NLRP3: NOD-, LRR-, and pyrin domain-containing protein 3
NNV: nervous necrosis virus
NP: nucleoprotein
NRF: nuclear respiratory factor
NS: non-structural protein
OMM: outer mitochondrial membrane
OPA: optic atrophy protein
OPTN: optineurin
ORF: open reading frame
OXPHOS: oxidative phosphorylation
PARL: presenilin-associated rhomboid-like protein
PEDV: Porcine epidemic diarrhea virus
PGAM5: phosphoglycerate mutase family member 5
PGC-1: peroxisome proliferator-activated receptor γ
PINK1: PTEN-induced putative kinase 1
PPAR: peroxisome proliferator-activated receptor
PRMT1: protein arginine methyltransferase 1
PRR: pattern recognition receptor
PRRSV: porcine reproductive and respiratory syndrome virus
PRV: pseudorabies virus
PVF: prototype foamy virus
RAB: Ras-related protein
RABV: rabies virus
RBR: RING-between-RING
RHEB: Ras homolog enriched in brain
RIG-1: retinoic acid inducible gene 1
RLR: RIG-1-like receptor
ROS: reactive oxygen species
RSV: respiratory syncytial virus
RVA: rotavirus A
XDY: Xijiao Dihuang decoction combined with Yinqiao powder
SAR-CoV-2: Severe acute respiratory syndrome coronavirus 2
SFN: Sulforaphane
SIAH1: seven in absentia homolog 1
SIRT: Sirtuin
STAT: signal transducer and activator of transcription
STING: stimulator of interferon genes
TAZ: transcriptional co-activator with PDZ-binding motif
TBK1: TANK-binding kinase 1
TFAM: transcription factor A, mitochondrial
TOMM: translocase of OMM
TLR: Toll-like receptor
TMD: transmembrane domain
TPR: tetratricopeptide repeat
TRAFs: TNF receptor-associated factors
TRIM: tripartite motif-containing
TRQ: Tanreqing
TUFM: Tu translation elongation factor
UBD: ubiquitin-binding domain
UBL: ubiquitin-like
ULK: UNC-51-like kinase
VDAC1: voltage-dependent anion channel protein 1
vIRF1: viral interferon regulatory factor 1
VMP1: vacuole membrane protein 1
VZV: Varicella-zoster virus
WHSC1L1: Wolf-Hirschhorn syndrome candidate 1-like 1
YAP: Yes-associated protein
ZIKV: Zika virus
